# Morphological, Toxicological, and Biochemical Characterization of Two Species of *Gambierdiscus* from Bahía de La Paz, Gulf of California

**DOI:** 10.3390/md22090422

**Published:** 2024-09-16

**Authors:** Leyberth José Fernández-Herrera, Erick Julián Núñez-Vázquez, Francisco E. Hernández-Sandoval, Daniel Octavio Ceseña-Ojeda, Sara García-Davis, Andressa Teles, Marte Virgen-Félix, Dariel Tovar-Ramírez

**Affiliations:** 1 Laboratorio de Toxinas Marinas y Aminoácidos, Centro de Investigaciones Biológicas del Noroeste, Av. Instituto Politécnico Nacional 195, Playa Palo de Santa Rita, La Paz CP 23096, Mexico; lfernandez@pg.cibnor.mx (L.J.F.-H.); dcesena04@cibnor.mx (D.O.C.-O.); 2 Laboratorio de Microalgas Nocivas, Centro de Investigaciones Biológicas del Noroeste, Av. Instituto Politécnico Nacional 195, Playa Palo de Santa Rita, La Paz CP 23096, Mexico; fhernan04@cibnor.mx; 3 Instituto Universitario de Bio-Orgánica Antonio González (IUBO AG), Universidad de La Laguna (ULL), Avenida Astrofísico Francisco Sánchez 2, 38206 La Laguna, Tenerife, Spain; sgdavis@ull.edu.es; 4 Laboratorio de Fisiología Comparada y Genómica Funcional, Centro de Investigaciones Biológicas del Noroeste, Av. Instituto Politécnico Nacional 195, Playa Palo de Santa Rita, La Paz CP 23096, Mexico; ateles@pg.cibnor.mx (A.T.); dtovar04@cibnor.mx (D.T.-R.); 5 Laboratorio de Colección de Microalgas, Centro de Investigaciones Biológicas del Noroeste, Av. Instituto Politécnico Nacional 195, Playa Palo de Santa Rita, La Paz CP 23096, Mexico; marte04@cibnor.mx

**Keywords:** *Gambierdiscus*, amino acids, pigments, toxicity, ciguatoxins, ciguatera, Gulf of California

## Abstract

We describe five new isolates of two *Gambierdiscus* species from Bahía de La Paz in the southern Gulf of California. Batch cultures of *Gambierdiscus* were established for morphological characterization using light microscopy (LM) and scanning electron microscopy (SEM). Pigment and amino acid profiles were also analyzed using high-performance liquid chromatography (HPLC-UV and HPLC-DAD). Finally, toxicity (CTX-like and MTX-like activity) was evaluated using the *Artemia salina* assay (ARTOX), mouse assay (MBA), marine fish assay (MFA), and fluorescent receptor binding assay (fRBA). These strains were identified as *Gambierdiscus* cf. *caribaeus* and *Gambierdiscus* cf. *carpenteri.* Toxicity for CTX-like and MTX-like activity was confirmed in all evaluated clones. Seven pigments were detected, with chlorophyll *a*, pyridine, Chl2, and diadinoxanthin being particularly noteworthy. For the first time, a screening of the amino acid profile of *Gambierdiscus* from the Pacific Ocean was conducted, which showed 14 amino acids for all strains except histidine, which was only present in *G.* cf. *caribeaus*. We report the presence of *Gambierdiscus* and *Fukuyoa* species in the Mexican Pacific, where ciguatera fish poisoning (CFP) cases have occurred.

## 1. Introduction

Ciguatera fish poisoning (CFP) is the most prevalent marine toxin-related seafood poisoning worldwide, affecting up to 50,000 people annually. This illness is caused by the consumption of seafood contaminated with the lipophilic toxins ciguatoxins (CTXs) and, possibly, maitotoxins (MTXs), which are produced by benthic dinoflagellates of the genera *Gambierdiscus* and *Fukuyoa* [1,2]. Numerous fish species that are typically associated with coral reefs worldwide accumulate CTXs from these dinoflagellates through the consumption of contaminated prey, storing the toxins in their flesh and viscera [1]. In tropical and subtropical regions, CFP is a serious problem that impacts the fishing industry and human health. The true incidence rate of CFP is challenging to determine, given that CFP-related symptoms are underreported (2–10%) [1,2]. The symptoms of CFP are characterized by gastrointestinal, neurological, and cardiovascular disorders and have been described as a polymorphic syndrome, with more than 175 reported symptoms and clinical signs. Symptoms can persist for days, months, or even years, and in severe cases, CFP can lead to paralysis, coma, and death [2].

Recent reviews have described an increase in the frequency and intensity of harmful algal blooms in some regions of the world [3,4,5,6]. These trends can be partly attributed to improved monitoring and coastal eutrophication [3,7,8]. Despite being significantly underreported, cases of CFP are increasing worldwide, with reports indicating a 60% rise in the Pacific Islands over recent decades and in other regions [1,9]. Climate variability may be related to the incidence of CFP [1,9,10] and Gingold et al. [10], with some research indicating that climate change could increase the burden of this poisoning.

In Mexico, CFP primarily occurs in the Yucatan Peninsula (PYUC) in the Mexican Caribbean, the Gulf of Mexico, and the waters of the southern Baja California Peninsula (PBC) in the Eastern Tropical Pacific and Gulf of California [11]. In the PBC, CFP cases are rare and sporadic; however, at least 240 cases (some severe) have been reported in the southern region due to the consumption of carnivorous fish, such as snappers (*Lutjanus* spp.) and cabrillas and groupers (*Epinephelus* and *Mycteroperca*), from islands and islets [11,12,13,14,15,16,17].

*Gambierdiscus* is a benthic dinoflagellate that grows on a variety of seagrasses, macroalgae, corals, artificial substrates, and sediments. Until 1999, *Gambierdiscus* was considered a monotypic genus (*G. toxicus*); however, in recent decades, 19 species have been described in this genus (*G. toxicus*, *G. australes*, *G. balechii*, *G. belizeanus*, *G. caribaeus*, *G. carolinianus*, *G. carpenteri*, *G. cheloniae*, *G. excentricus*, *G. holmesii*, *G. honu*, *G. jejuelnsis*, *G. lapillus*, *G. lewissi*, *G. pacificus*, *G. polynensiensis*, *G. scabrosus*, *G. silvae*, and *G. vietnamensis*) [18]. *Fukuyoa* is closely related to *Gambierdiscus* and was originally included in this genus [18,19,20] before being reclassified by Gomez et al. [20]. Four species of *Fukuyoa* have been described: *F. yasumutoi*, *F. ruetzleri*, *F. paulensis*, and *F. koreensis* [20,21]. 

*Gambierdiscus* is widely distributed in coastal zones at tropical and subtropical latitudes; however, its presence has also been reported in temperate waters, including those near Australia, Japan, and the Mediterranean [22]. The breadth of species within this genus remains poorly studied, and the ability to discriminate between different *Gambierdiscus* species has only recently been developed [23]. *Gambierdiscus* produces multiple secondary metabolites, including bioactive compounds that can be either lipophilic or hydrophilic and vary in their modes of action and toxicity. These include CTXs, MTXs, gambierones, gambieroles, gambieroxide, and gambieric acids [18,22]. To date, more than 30 CTX congeners have been identified. 

Ciguatoxins are lipophilic, ladder-shaped polyethers with 13–14 cyclic consecutively connected rings [22] and are classified as either CTX3C, Caribbean Sea CTXs (C-CTXs), Pacific Ocean CTXs (P-CTXs/CTX4A), or Indian Ocean CTXs (I-CTXs) based on the structural backbone of each molecule [22]. Recently, a new ciguatoxin was isolated from *G. silvae* and *G. caribeus* in the Caribbean; its structure was determined, and it was named C-CTX-5 [24,25]. Although some MTXs exhibit greater toxicity than CTXs, their role in CFP remains unclear. Six MTXs have been identified, and most of their structures have been elucidated. Like CTXs, MTXs are also polyether compounds, but their molecular masses vary significantly. Most MTXs are larger than CTXs, although they share structural similarities [22]. In addition to CTXs and MTXs, some *Gambierdiscus* species produce gambieric acids (GA), gambieroxide, gambierol, and gambierons. Some of these compounds have antifungal properties and participate in dinoflagellate defense mechanisms while also possibly regulating their growth. They also exhibit substantially lower toxicity than CTXs, opening the door for potential therapeutic and biomedical applications [22,26].

Evaluating the pigment profile, amino acids, and other metabolites in dinoflagellate species provides an opportunity to explore the chemical diversity of secondary metabolites. These metabolites can serve as biomarkers for identifying *Gambierdiscus* species (chemotaxonomy); they can also be used to evaluate possible ecotypes and to study toxin metabolism and biosynthesis, which may potentially involve allelochemical compounds [27,28].

*Gambierdiscus toxicus*, *Gambierdiscus* sp., *F. yasumotoi*, *G.* cf. *carolinianus*, *G.* cf. *carpenteri*, *Gambierdiscus* spp., and *Fukuyoa* sp. have been documented in the Eastern Tropical Pacific and Gulf of California around the southern PBC [29,30,31,32,33,34,35,36,37,38] (Table S1). In this work, we describe five new strains of *G.* cf. *caribaeus* and *G.* cf. *carpenteri* from Bahía de La Paz in the southern Gulf of California. We propose a morphological characterization using light microscopy (LM) and scanning electron microscopy (SEM) and report pigment and amino acid profiles using high-performance liquid chromatography and ultraviolet-visible spectrophotometry (HPLC-UV/VIS) and high-performance liquid chromatography-diode array detection (HPLC-DAD). Toxicity (CTXs-like and MTXs-like activity) was evaluated by four different assays: *Artemia salina* assay (ARTOX), mouse bioassay (MBA), marine fish assay (MFA), and fluorescent receptor binding assay (fRBA).

## 2. Results

### 2.1. Isolation and Algal Cultures

*Gambierdiscus* was present at all sampling sites in Bahía de La Paz (Table S2). Seventy-five percent of isolated cells were established in culture (n = 39 well plate); the lenticular cells were isolated directly from the substrate without shaking the algae or sonicating the sediment. 

### 2.2. Morphological Identification

**Figure 1:**
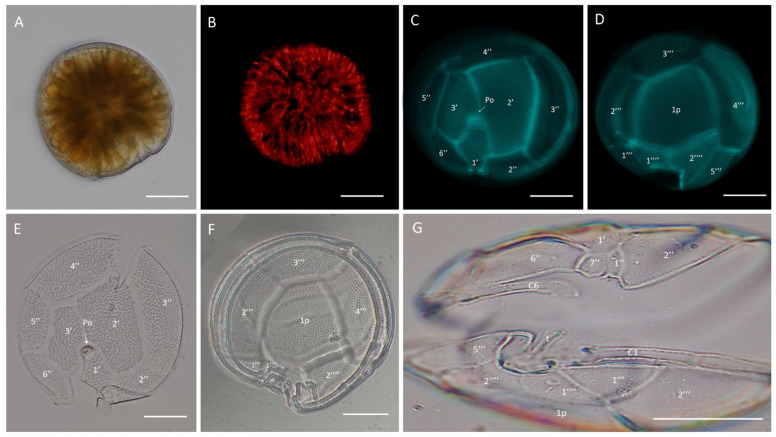
Light microscopy of *Gambierdiscus* cf. *caribaeus* (GbSa-7). (**A**) Live cell, (**B**) Epifluorescence of chloroplast, (**C**) Plate tabulation apical view stained with Fluorescent Brightener 28, (**D**) Antapical view, (**E**) Apical view, (**F**) Antapical view, and (**G**) Ventral view. Scale bars: (**A**–**F**) 50 µm and (**G**) 30 µm.

*Gambierdiscus* cf. *caribaeus* strains GbSa-2 and GbSa-7 were identified as photosynthetic, lens-shaped cells that were rounded in the apical view and compressed in the anterior-posterior axis, with golden granular chloroplasts (Figure 1A,B). The cells measured 70.31–91.61 ± 5.90 µm depth, 83.32–86.32 ± 7.90 µm width, and 61.12–64.57 ± 4.10 µm length (n = 30). The plate formula was Po, 3′, 7″, 6C, 6S, 5′′′, 1p, and 2′′′′ (Figure 1C–F). The apical pore plate Po was in contact with apical plate 1′ 2′, and 3′. The rectangular hatched-shaped 2′ plate was the largest apical plate, with plate 4″ being pentagonal and symmetrical (Figure 1C,E). In the antapical view, between 2′′′ and 4′′′, plate 1p was the largest hypothecal plate; it was broad and pentagonal in shape. In the ventral view, minuscule plate 1″ was rectangular, and trapezium-shape 7″ was also observed (Figure 1G). 

**Figure 2:**
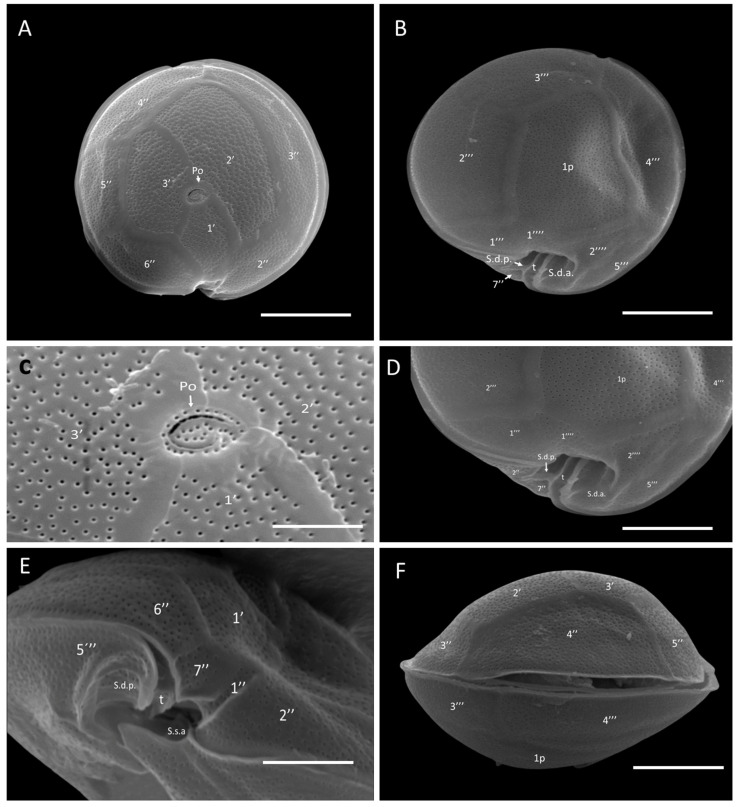
Scanning electron micrographs of *Gambierdiscus* cf. *caribaeus* (GbSa-7). (**A**) Apical view, (**B**) Antapical view, (**C**) Po plate, (**D**) Sulcal area in antapical view, (**E**) Sulcal area in ventral view, and (**F**) Dorsal view. Scale bars: (**A**,**B**) 50 µm, (**C**) 10 µm, (**D**) 20 µm, and (**E**,**F**) 30 µm.

SEM micrographs (n = 15) revealed a rounded apical shape with a smooth cell surface and multiple pores scattered across the thecal plates, including symmetrical plate 4″ (Figure 2A). The antapical view showed a broad 1p plate and detail of postcingular plates 1′′′, 2′′′, 3′′′, and 4′′′ (Figure 2B,D). The apical pore plate, Po, was elliptical with a fishhook-shaped opening (Figure 2C). Detailed images of the sulcal zone showed minuscule plates 1″ and 7″, with the cingulum gradually descending near the sulcus (Figure 2E). The dorsal view revealed symmetrical plate 4″ and a narrow, stylized cingulum edge (Figure 2F). Both LM and SEM micrographs of the GbSa-2 strain are shown in Figure S3. 

The maximum cell density (1835 ± 117 cells mL^−1^) in *G.* cf. *caribaeus* was recorded during the exponential phase (day 35 for strain GbSa-2). For strain GbSa-7, the maximum cell density (2457 ± 394 cells mL^−1^) was recorded on day 49. The calculated growth rates were 0.25 ± 0.01 and 0.26 ± 0.05 division·day^−1^, respectively, with an average generation time of 2.71 ± 0.06 days for *G.* cf. *caribaeus* strains.

**Figure 3:**
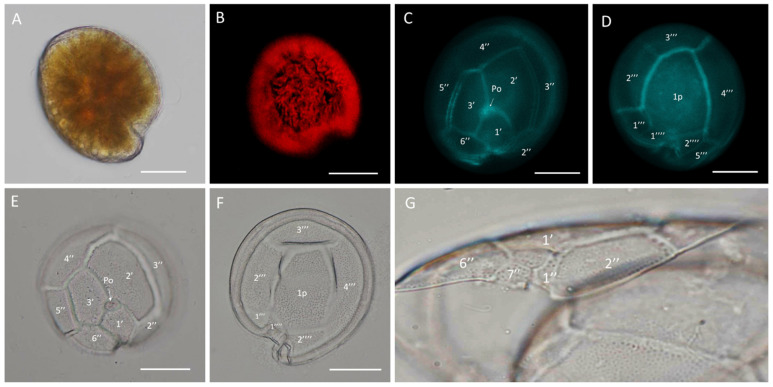
Light microscopy of *Gambierdiscus* cf. *carpenteri* (GbSa-4). (**A**) Live cell, (**B**) Epifluorescence of chloroplast, (**C**) Plate tabulation apical view stained with Fluorescent Brightener 28, (**D**) Antapical view, (**E**) Apical view, (**F**) Antapical view, and (**G**) Ventral view. Scale bars: (**A**–**F**) 50 µm and (**G**) 30 µm.

In *G.* cf. *carpenteri* strains GbSa-4, GbSa-5, and GbSa-7, the cells were photosynthetic and lens-shaped, predominantly oblong and irregular in apical view, and compressed in the anterior-posterior axis, with golden granular chloroplasts (Figure 3A,B). The cells measured 72.21–84.91 ± 5.90 µm depth, 71.72–77.62 ± 7.29 µm width, and 59.56–64.57 ± 6.15 µm length (n = 30). The plate formula was Po, 3′, 7″, 6C, 6S, 5′′′, 1p, and 2′′′′ (Figure 3C–F). 

In the apical view, plates 1′, 2′, and 3′ were connected to the Po plate. Apical 2′ plate showed a hatched and large form, while plate 4″ was asymmetrically narrow toward plate 3″ and wider toward plate 5″. Plates 2″ and 6″ were the shortest in the precingular area (Figure 3C,E). Plate 1p was long, pentagonal, and broad, joining with small plates 1′′′′ and 2′′′′ in a ridge-like form. Plate 1p was also connected to plate 3″ in a quadrangular shape and to plates 2′′′ and 4′′′ to form the largest quadrangular plate (Figure 3D,F). In the ventral view, minuscule plate 1″ was rectangular and plate 7″ was trapezium-shaped (Figure 3G). 

**Figure 4:**
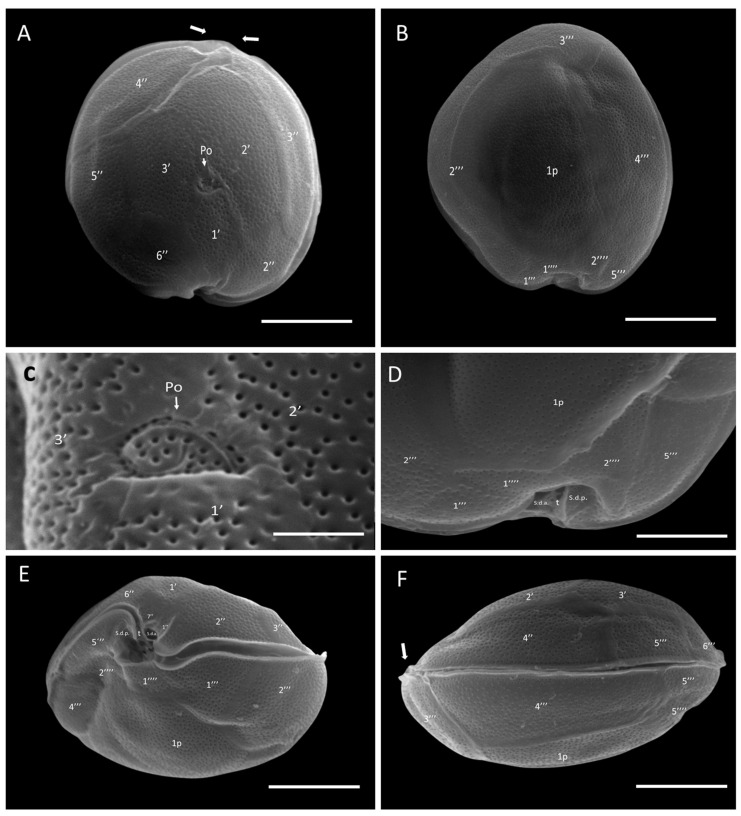
SEM micrographs of *Gambierdiscus* cf. *carpenteri* (GbSa-4). (**A**) Apical view arrow showing an apparent rostrum, (**B**) Antapical view, (**C**) Po plate, (**D**) Sulcal area in antapical view, (**E**) Sulcal area in ventral view, and (**F**) Dorsal view arrow points to apparent rostrum. Scale bars: (**A**,**B**) 50 µm, (**C**) 10 µm, (**D**) 20 µm, and (**E**,**F**) 30 µm.

SEM micrographs (n = 15) of *G.* cf. *carpenteri* revealed a rounded apical shape with a smooth cell surface and multiple scattered pores. Asymmetrical plate 4″ protruded, forming an apparent rostrum where plate 3″ was articulated (Figure 4A). The antapical view revealed a long, pentagonal plate 1p (Figure 4B,D). The apical pore plate, Po, was elliptical with a fishhook-shaped opening (Figure 4C). The sulcal zone showed minuscule plate 1″ and small plate 7″, which formed ridges toward the adjacent part of the sulcus (Figure 4E). The cingulum was narrow, and the dorsal view highlighted asymmetrical 4″ plate and the apparent rostrum formed by the union of precingular 3″ and 4″ plates and postcingular 3′′′ and 4′′′ plates (Figure 4F). The LM and SEM micrographs of GbSa-5 and GbSa-6 strains are shown in Figures S4 and S5.

*Gambierdiscus* cf. *carpenteri* strains GbSa-4 and GbSa-6 presented higher cellular densities (2779 ± 424 and 1815 ± 329 cells mL^−1^, respectively) compared to that of strain GbSa-5 (892 ± 163 cells mL^−1^). The growth rates, based on cell counts for the strains with the highest abundance, were 0.25–0.27 ± 0.01 division·day^−1^, with an average generation time of 2.64 ± 0.09 per day. Strain GbSa-5 showed the lowest growth rate among all strains (0.21–0 ± 0.01 division·day^−1^, 3.36 ± 0.20 generation time). The growth curves are shown in Figure S6.

Morphological measurements revealed differences in the length-to-width ratios of *G.* cf. *caribaeus* cell size (1.05–1.13 ± 0.59 and 1.00–1.12 ± 1.29) compared to that of *G.* cf. *carpenteri*. The measurements of the apical pore plate were similar between the strains of both species, although the number of pores differed. *Gambierdiscus* cf. *caribaeus* and *G.* cf. *carpenteri* exhibited an average of 37.33 ± 3.77 pores and 40.66 ± 2.43 pores, respectively. The length-to-width ratio of plate 1p further distinguished the two species (*G.* cf. *caribaeus*: ratio of 1.28 ± 0.11; *G.* cf. *carpenteri*: 1.59 ± 0.24; Table 1).

Regarding the ratio of the 2′/4″ suture length and 2′/2″ suture length between the two species, no differences were observed in the measurements (n = 20). In *G.* cf. *caribaeus*, this ratio was 0.92 ± 0.01, while in *G.* cf. *carpenteri*, the ratio was 0.92 ± 0.04. After the dissociation of the apical and antapical plates, marked morphological differences were only observed in plates 1′, 3″, and 4″.

In addition to the dinoflagellates characterized in this study, we also detected the presence of the potentially harmful dinoflagellates *G.* cf. *carolineanus*, *G.* cf. *toxicus*, and *F.* cf. *yasumotoi* (Figures S7–S9). 

### 2.3. Biochemical Characterization

#### 2.3.1. Pigments Composition

**Figure 5:**
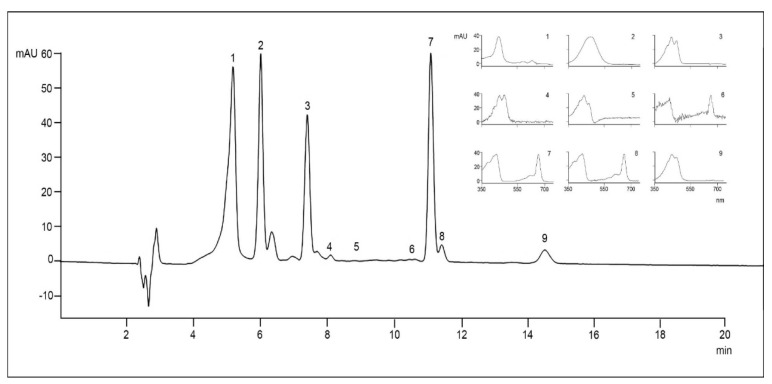
To investigate the composition and concentration of pigments, samples were collected between days 24 and 25 of culture during the early exponential growth phase. As a result of this, nine pigments were identified in *Gambierdiscus* strains (Figure 5 and Figure 6).

**Figure 6:**
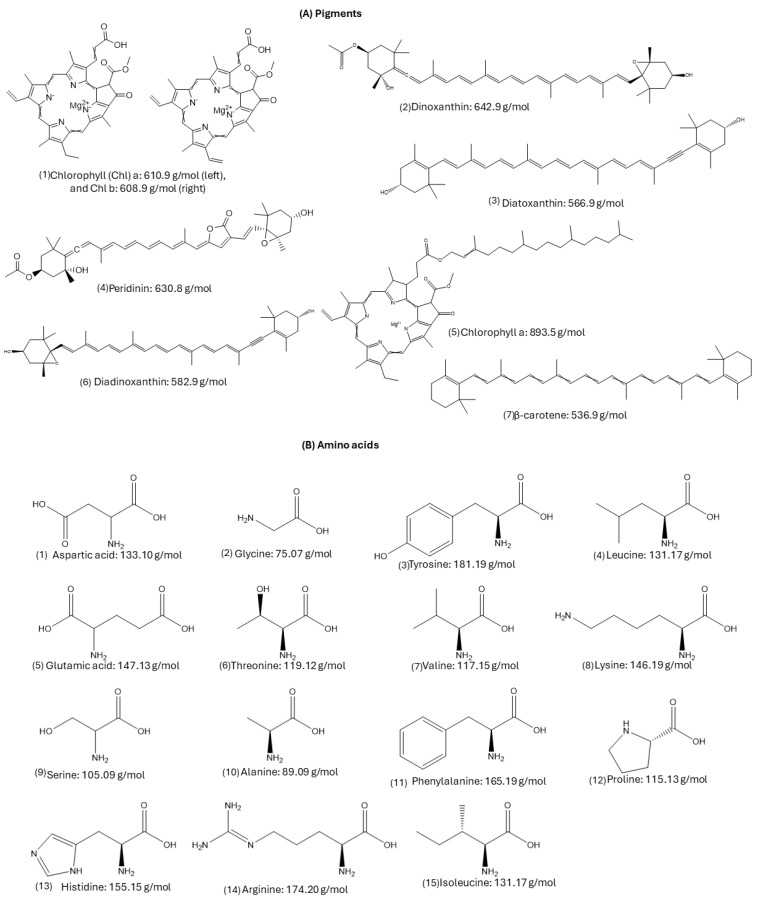
Principal pigments and amino acids detected in five strains of *Gambierdiscus* cf. *caribaeus* and *G.* cf. *carpenteri* in this study. (**A**) Pigments: (1) chlorophyll *c*1-*c*2, (2) Dinoxanthin, (3) diatoxanthin, (4) peridinin, (5) Chlorophyll a, (6) diadinoxanthin, and (7) β, β-carotene. (**B**) Amino acids: (1) aspartic acid, (2) glycine, (3) tyrosine, (4) leucine, (5) glutamic acid, (6) threonine, (7) valine, (8) lysine, (9) serine, (10) alanine, (11) phenylalanine, (12) proline, (13) histidine, (14) arginine, and (15) isoleucine.

To investigate the composition and concentration of pigments, samples were collected between days 24–25 of culture during the early exponential growth phase. As a result of this, nine pigments were identified in *Gambierdiscus* strains (Figure 5 and Figure 6). 

Pigment production varied between the strains (Table 2). Peridinine was the main carotenoid pigment present in the *Gambierdiscus* strains (23.89–81.26 pg cell^−1^). Chlorophyll *a* was the major pigment in *G*. cf. *carpenteri* GbSa-6 (278.9 pg·cell^−1^), followed by chlorophyll *c*1, *c*2 (57.59 pg·cell^−1^) in *G.* cf. *caribaeus* GbSa-2.

#### 2.3.2. Amino Acid Composition

**Figure 7:**
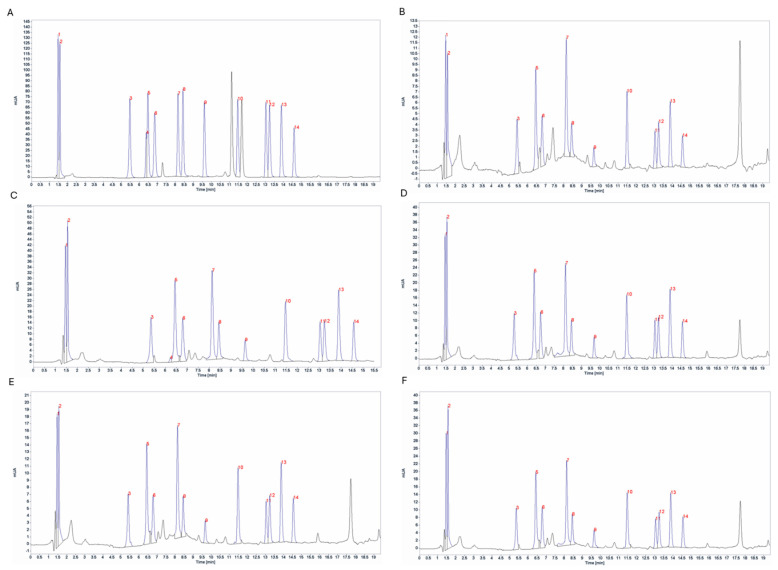
High-performance liquid chromatography (HPLC) chromatograms registered at 338 nm. (**A**) STD amino acids. Peak identification: (1) aspartic acid, (2) glutamic acid, (3) serine, (4) histidine, (5) glycine, (6) threonine, (7) alanine, (8) arginine, (9) tyrosine, (10) valine, (11) phenylalanine, (12) isoleucine, (13) leucine, and (14) lysine. *G.* cf. *caribaeus* strain (**B**) Gbsa-2, (**C**) Gbsa-7, *G.* cf. *carpenteri* strain (**D**) Gbsa-4, (**E**) Gbsa-5 and (**F**) Gbsa-6. The chromatograms for proline are shown in Figure S10.

The amino acid profile of *Gambierdiscus* was determined using HPLC-DAD. Fourteen amino acids were detected, including glutamic acid, aspartic acid, serine, glycine, threonine, alanine, arginine, tyrosine, valine, phenylalanine, isoleucine, leucine, lysine, and proline. Histidine was detected only in *G.* cf. *caribaeus* Gbsa-7 (Figure 7).

The amino acid content varied between the strains (Table 3). Glutamic acid had the highest content (14.80–36.39 pg·cell^−1^), whereas glycine had the lowest (7.72–18.66 pg·cell^−1^). Strain GbSa-7 contained proline (<52.30 pg·cell^−1^) and was the only strain to contain histidine.

### 2.4. Toxicity Assay

#### 2.4.1. Mouse Bioassay (MBA)

All *Gambierdiscus* strains tested by MBA were positive for CTX-like activity. The toxicity in *G.* cf. *caribaeus* GbSa-2 was 18.57 × 10^−4^ µg·kg^−1^·1000 cells^−1^, which was comparable to the toxicity of strain GbSa-7 (17.08 × 10^−4^ µg·kg^−1^·1000 cells^−1^). For *G.* cf. *carpenteri* strains, significant variations were observed. Strain GbSa-4 showed the highest toxicity (29.07 × 10^−4^ µg·kg^−1^·1000 cells^−1^), with CTXs-like activity three times higher compared to that of strain GbSa-5 (8.46 × 10^−4^ µg·kg^−1^·1000 cells^−1^) and more than twice that of strain GbSa-6 (12.54 × 10^−4^ µg·kg^−1^·1000 cells^−1^).

**Figure 8:**
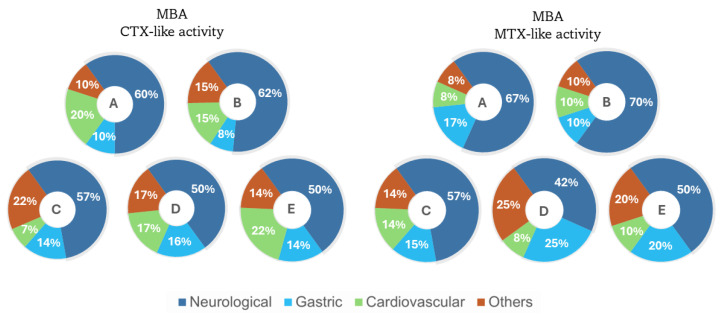
Left ring charts showed clinical signs recorded during the mouse bioassay (MBA) using CTX-like activity for *Gambierdiscus* cf. *caribaeus* strains (A) GbSa-2 and (B) GbSa-7 and *Gambierdiscus* cf. *carpenteri* strains (C) GbSa-4, (D) GbSa-5, and (E) GbSa-6. Right ring chart clinical signs recorded during the MBA using MTX-like activity of *Gambierdiscus* cf. *caribaeus* strains (A) GbSa-2 and (B) GbSa-7) and *Gambierdiscus* cf. *carpenteri* strains (C) GbSa-4, (D) GbSa-5, and (E) GbSa-6.

The main clinical signs related to CTXs-like activity were neurological: paralysis of the rear and front quarters, dyspnea, piloerection, spasms, loss of locomotion, body arching, arched and zig-zag tail movements, tail wriggling, blinking, and ataxia. Gastric signs included polyuria, lethargy due to peritoneal inflammation, diarrhea, and abundant defecation. Cardiovascular signs included labored breathing and vasoconstriction of the ear and other clinical signs like continuous itching and scratching, hypersalivation, closed eyes, and eventual death due to respiratory failure (Figure 8).

All strains of *G.* cf. *caribaeus* and *G.* cf. *carpenteri* showed toxicity related to MTX-like activity in the MBA at sublethal doses. After 24 h of observation, the LD_50_ was not recorded in any of the groups of injected mice, but several clinical signs related to MTX-like activity were observed.

The clinical signs associated with MTXs-like activity included neurological signs such as paralysis of the rear and front quarters, dyspnea, piloerection, spasms, body arching, arched tail and tail wriggling, blinking, ataxia, and convulsions. Gastric signs included polyuria, lethargy due to peritoneal inflammation, and abundant defecation. Cardiovascular signs included vasoconstriction of the ears. Other clinical signs included hypersalivation, closed eyes, and tear secretion (Figure 8). 

#### 2.4.2. *Artemia* Assay (ARTOX) 

**Figure 9:**
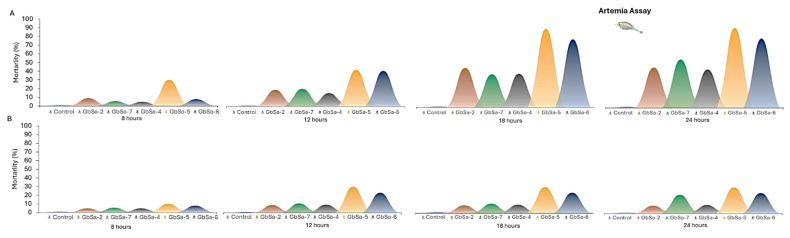
*Artemia salina* lethality bioassay. (**A**) Brine shrimp exposed to CTX-like activity of *Gambierdiscus* cf. *caribaeus* (GbSa-2 and GbSa-7) and *Gambierdiscus* cf. *carpenteri* (GbSa-4, GbSa-5, and GbSa-6). (**B**) Brine shrimp exposed to MTX-like activity of *Gambierdiscus* cf. *caribaeus* (GbSa-2 and GbSa-7) and *Gambierdiscus* cf. *carpenteri* (GbSa-4, GbSa-5, and GbSa-6).

Toxicity was determined using the brine shrimp *Artemia salina*. The control group did not exhibit any mortality, whereas the organisms exposed to CTXs-like activity of *Gambierdiscus* strains showed mortality (Figure 9) and clinical signs of toxicity. 

At 6 h, the mortality of the brine shrimp exposed to the CTXs-like activity of *G.* cf. *caribaeus* strains GbSa-2 and GbSa-7 was 9 ± 1% and 5 ± 0.5%, respectively. The mortality caused by *G.* cf. *carpenteri* were 5 ± 1% for strain GbSa-4, 30 ± 3% for strain GbSa-5, and 8 ± 1% for strain GbSa-6. 

After 12 h, *G.* cf. *caribaeus* GbSa-2 caused 18 ± 1% mortality, while strain GbSa-7 caused 14 ± 1%. The mortalities caused by *G.* cf. *carpenteri* in brine shrimp were 19 ± 1% for strain GbSa-4, 40 ± 3% for strain GbSa-5, and 39 ± 2% for strain GbSa-6.

The highest mortality in *A. salina* was recorded at 18 h of exposure to the CTXs-like activity of *G.* cf. *caribaeus* GbSa-2 (45 ± 3%) and GbSa-7 (38 ± 2%). For the *G.* cf. *carpenteri* extract, mortality was 38 ± 1% for strain GbSa-4, 90 ± 6% for strain GbSa-5, and 78 ± 5% for strain GbSa-6. This trend remained consistent until 24 h when the mortality of *A. salina* was 45 ± 3% for GbSa-2 and 43 ± 2% for GbSa-7. The highest mortality was observed for *G.* cf. *carpenteri* GbSa-5 (90 ± 12%), followed by GbSa-6 (78 ± 5%) and GbSa-4 (54 ± 3%) (Figure 9A). 

Clinical signs in *A. salina* exposed to CTX-like activity were characterized by changes in swimming behavior. Starting at 6 h, *A. salina* exposed to *G*. cf. *caribaeus* and *G.* cf. *carpenteri* showed a loss of orientation while swimming, characterized by swimming in circles and rising and falling from the surface to the bottom. These clinical signs intensified between 6 and 24 h, leading to gasping, lethargy, and paralysis of the thoracic appendages.

For *A. salina* exposed to MTX-like activity, mortality was recorded at 6 h: 5 ± 0.5% and 5 ± 1% for strains GbSa-2 and GbSa-7 of *G.* cf. *caribaeus* and 5 ± 0.5%, 10 ± 1%, and 8 ± 5% for strains GbSa-4, GbSa-5, and GbSa-6 of *G.* cf. *carpenteri*, respectively. After 12 h of exposure to the MTX-like activity of *G.* cf. *caribaeus*, the mortality of *A. salina* was 9 ± 1% (GbSa-2) and 11 ± 1% (GbSa-7). At this time, the highest mortality was recorded with GbSa-5 (30 ± 4%) and GbSa-6 (23 ± 2%) from *G.* cf. *carpenteri*, while GbSa-4 showed 10 ± 2% mortality. At 18 h, *A. salina* mortality did not increase further in any of the treatments. At 24 h, *A. salina* mortality increased by 10% for those exposed to the MTXs-like activity of *G.* cf. *caribaeus* GbSa-7 (<22 ± 2%) (Figure 9B).

Although mortality due to MTX-like activity in *A. salina* was lower compared to that of CTX-like activity, several clinical signs were present from the first hour of exposure. Brine shrimp exhibited erratic swimming, lethargy, a head-up posture, reduced response to stimuli, and gasping within the first 12 h. By the end of the exposure, 80% of brine shrimp had recovered.

#### 2.4.3. Marine Fish Assay

**Figure 10:**
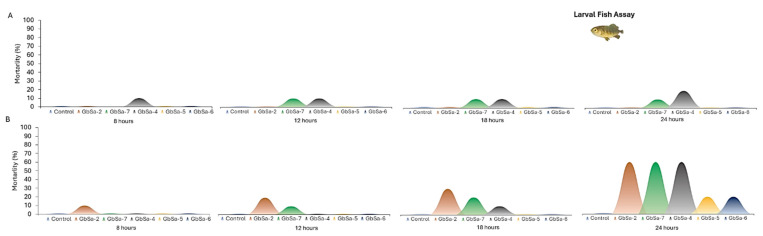
(**A**) Toxicity in *Seriola rivoliana* larvae exposed to the CTX-like activity of *Gambierdiscus* cf. *caribaeus* (GbSa-2 and GbSa-7) and *Gambierdiscus* cf. *carpenteri* (GbSa-4, GbSa-5, and GbSa-6). (**B**) Toxicity in larvae of *Seriola rivoliana* exposed to MTX-like activity of *Gambierdiscus* cf. *caribaeus* (GbSa-2 and GbSa-7) and *Gambierdiscus* cf. *carpenteri* (GbSa-4, GbSa-5, and GbSa-6).

A toxicity test was conducted using longfin yellowtail larvae (*Seriola rivoliana*). During the first 6 h, only larvae exposed to the CTX-like activity of *G.* cf. *carpenteri* GbSa-4 presented 10 ± 1% mortality. After 12 h of treatment, *G.* cf. *caribaeus* GbSa-7 caused 10 ± 0.5% mortality. At 18 h, there was no further increase in mortality in any of the treatments. The maximum mortality was recorded at 24 h, with 20 ± 2% mortality in *S. rivoliana* larvae exposed to the CTXs-like activity of *G.* cf. *carpenteri* GbSa-4. No mortality was observed in the control group (Figure 10A).

Clinical signs recorded in *S. rivoliana* larvae exposed to CTXs-like activity included a loss of balance with abnormal horizontal orientation, abnormal swimming behavior, loss of buoyancy control leading to sinking to the bottom, reduced response to stimuli, erratic swimming, shaking, body arching, irregular ventilation (e.g., hyperventilation), and notable skin darkening.

Larvae exposed to the MTXs-like activity of *G.* cf. *caribaeus* GbSa-2 exhibited 10 ± 1% mortality at 6 h, which increased to 20 ± 2% at 12 h. Larvae exposed to the MTX-like activity of *G.* cf. *caribaeus* GbSa-7 showed 10 ± 1% mortality at 6 h. At 18 h, mortality due to GbSa-2 and GbSa-7 increased to 30 ± 3%. In contrast, larvae treated with the MTXs-like activity of *G.* cf. *carpenteri* GbSa-4 showed 10 ± 0.5% mortality. After 24 h, the maximum mortality of *S. rivoliana* larvae was 60 ± 4% for those exposed to the MTXs-like activity of *G.* cf. *caribaeus* GbSa-2 and GbSa-7 and *G.* cf. *carpenteri* GbSa-4. The lowest mortality was observed in larvae exposed to the MTXs-like activity of *G.* cf. *carpenteri* GbSa-5 and GbSa-6, with 20 ± 1% mortality (Figure 10B).

The exposure of larvae to dissolved MTX-like activity led to abnormal horizontal orientation, loss of balance, and loss of buoyancy control, causing the larvae to sink to the bottom. Abnormal vertical orientation with head-down posture was also observed, along with behavioral changes such as erratic swimming and pulses of hypoactivity and hyperactivity. Additional signs included shaking, body arching, abnormal respiration (e.g., hyperventilation), irregular ventilation, head shaking, and skin darkening before death. 

### 2.5. Fluorescent Receptor Binding Assay (fRBA)

To measure the binding affinity of the CTX analogs for voltage-gated sodium channel (VGSC) receptors, we employed the fRBA assay. This assay is based on the competition for VGSC site **5** between CTXs and a brevetoxin (PbTx) labeled with fluorescent compounds. The relative fluorescence unit (RFU), defined as the fluorescence relative to the culture biomass, was adjusted to the control (ethanol 100% binding value, representing maximum fluorescence). For the highest concentration of the standard curve, the PbTx standard at 0.1 µg·mL^−1^ showed 29.8 ± 4.95% RFU. The *Karenia brevis* (Kb-3) extract (positive for PbTx-2 and Pbtx-3) resulted in 95.15 ± 2.10% RFU, with a concentration of 1.3 ± 0.07 pg eq PbTx per cell. For *G.* cf. *caribaeus* GbSa-2, the blinding value was 95.6 ± 0.89% RFU with a maximum concentration of 2.59 ± 0.25 pg eq PbTx per cell. Strain GbSa-7 showed a binding value of 92.8 ± 1.01% RFU with a concentration of 2.19 ± 0.23 pg eq PbTx per cell.

The affinity for VGCS in *G.* cf. *carpenteri* GbSa-4 was 93.9 ± 1.27% RFU, with a maximum concentration of 2.51 ± 0.30 pg eq PbTx per cell. The highest competition for VGCS was recorded for strains GbSa-5 (89.8 ± 2.50% RFU and 1.15 ± 0.31 pg eq PbTx per cell) and GbSa-6 (95.8 ± 3.49% RFU and 1.57 ± 0.05 pg eq PbTx per cell).

## 3. Discussion

### 3.1. Morphological Identification

Two out of the five isolated strains were identified as *G.* cf. *caribaeus,* and three of them were identified as *G.* cf. *carpenteri*, as both species share similarities in the plate formula Po, 3′, 7″, 6C, 6S, 5′′′, 1p, and 2′′′′ (Figure 1, Figure 2, Figure 3 and Figure 4). However, they can be distinguished by the shape of plates 1p and 4″, as suggested by the dichotomous key of Litaker et al. [39] and the description of Diaz-Ascencio et al. [40]. *Gambierdiscus* cf. *caribaeus* (GbSa-2 and GbSa-7) is characterized by a rounded and lenticular-shaped cell, short and wide plate 1p, and symmetrical plate 4″, which is consistent with the original descriptions by Litaker et al. [39] and Soler-Onis et al. [41] in Atlantic waters and Diaz-Ascencio et al. [42] in the Caribbean region (Figure 2). Although *G. caribaeus* shares morphological features with its sister species, *G. carpenteri* and *G. jejuensis*, the presence of a symmetrical plate 4” allows for satisfactory differentiation. However, under culture conditions, this is not always reliable, as cellular deformation occurs during the initial generations, leading to aberrant cell shapes that complicate the identification of the typical plates characterizing the genus *Gambierdiscus* [40,42,43]. In this study, the number of deformed cells in *G.* cf. *caribaeus* strains was low, and the morphological features aligned with previous descriptions, suggesting that measurements were accurate.

Strains of *G.* cf. *carpenteri* (GbSa-4, GbSa-5, and GbSa-6) from the Gulf of California exhibited clear morphological characteristics similar to those described by Litaker et al. [39]. These included oval cells, a narrower and elongated plate 1p extending toward plate 3′′′′, and an asymmetric plate 4″ (Figure 3C–F). Cell size was similar to those of the strains reported for *G. carpenteri* isolated from Australia, Belize, the Mexican Caribbean, and the Philippines [39,44,45]. However, variations were observed in the shape of plate 2′, which ranged from rectangular to hatchet-shaped. Additionally, the *G.* cf. *carpenteri* strains described in this study presented several morphological characteristics similar to those described by Ramos-Santiago et al. [38] for *G. carpenteri* in the Gulf of California, with plates 2′ and 4″ corresponding to those described by Litaker et al. [39,44]. Furthermore, in the description by Litaker [39], *G. carpenteri* cells presented a rostrum resulting from the fusion of plates 3′ and 4″ and postcingular plates 3′′′ and 4′′′, which was similarly observed in *G.* cf. *carpenteri* (SEM GbSa-4 and GbSa-6, n = 14) (Figure 4A–F and Figure S6). However, the cellular morphology of *G.* cf. *carpenteri* (GbSa-4 and GbSa-6) was more consistent with the descriptions by Kohli et al. [44] and Ramos-Santiago et al. [38]. The variety of forms among the strains isolated from the same area supports the diversity and morphological plasticity of *G. carpenteri*, suggesting that the diverse coastal environments of the Gulf of California may harbor new ecotypes of this species.

**Figure 11:**
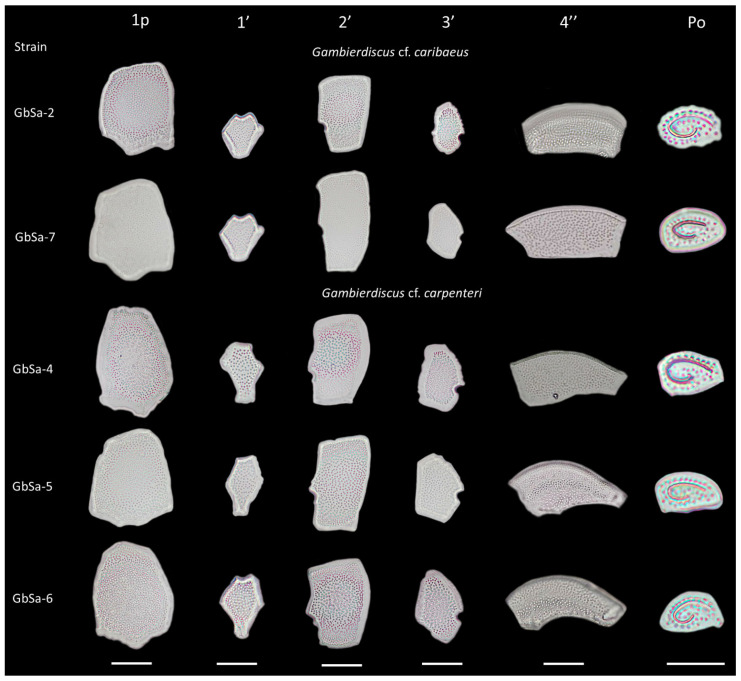
Light microscopy of typical morphology plate 1p, 1′, 2′, 3′, 4″, Po plate under present in *Gambierdiscus* cf. *caribaeus* (GbSa-2, GbSa-7) and *Gambierdiscus* cf. *carpenteri* (GbSa-4, GbSa-5, GbSa-6). Scale bar: 20 µm.

Plate dissociation allowed for precise measurements and detailed observations using LM, which confirmed the formula tabulation [39]. Through plate dissociation, it was possible to support the morphological distinction between *G.* cf. *caribaeus* and *G.* cf. *carpenteri* and to perform more precise measurements, particularly in plate 1p. Differences in the ratio were observed between *G.* cf. *caribaeus* and *G.* cf. *carpenteri* (Table 1), although the diversity in the shape of plate 2′ did not correspond to the ratio of the 2′/4″ suture length and 2′/2″ suture length between the two species, as suggested by Diaz-Asencio et al. [40], Vacarizas et al. [45], and Litaker et al. [39]. Plate dissociation was performed to compare the shape and size of plates 1′ and 3′, leading to the suggestion of adding the shape of plate 4″ to the tabulation to distinguish *G.* cf. *caribaeus* from *G.* cf. *carpenteri* (Figure 11). Plate 1′ was more compact and robust in *G.* cf. *caribaeus*, while plate 3′ was wider in *G.* cf. *carpenteri* than in *G.* cf. *caribaeus*, including in deformed cells (Figure 11). The asymmetric plate 4″ was similar in all *G.* cf. *carpenteri* strains, and upon dissociation, it was compared with fixed cells of *G. autrales* (VGO1250), *G. excentricus* (VGO911), and *G. carolineanus* (GbEs-4), which also exhibited an asymmetrical plate 4′. In *G.* cf. *carpenteri*, the width contrast in the 3″/4″ suture was twice as small as that in the 4″/5″ suture, whereas in *G. autrales*, *G. excentricus*, and *G. carolineanus,* the proportion was lower (1.5). Regarding the forms of plate Po, only the number of pores showed differences between *G.* cf. *caribaeus* and *G.* cf. *carpenteri*. Therefore, we recommend performing plate dissociation to morphologically distinguish *G. caribaeus*, *G. carpenteri*, and *G. carolineanus* (Figure 11 and Figure S7).

*Gambierdiscus caribeaus* and *G. carpenteri* are two species within the genus that present a cosmopolitan distribution [46]. They exhibit a wide range of tolerance to variations in temperature, salinity, and irradiance [44,46], which contributes to their broad distribution and ability to adapt to diverse habitats, as well as their efficient growth rates [47,48,49]. In this study, *G.* cf. *caribaeus* and *G.* cf. *carpenteri* showed the highest cell growth under culture conditions, with average growth rates of 0.25 div/day, similar to those reported in previous studies under optimal salinity (35) and temperature (25 °C) conditions [45,50]. However, *G.* cf. *carpenteri* strain GbSa-5 exhibited a slower growth rate and lower cell abundance compared with the GbSa-4 and GbSa-6 strains.

Although the toxicity levels of *G. carpenteri* are considered medium-to-low and their contribution to CFP is uncertain [46], it is important to consider the potential implications of the presence of this species in the Gulf of California. Various factors have been identified that could increase the risk of CFP cases [1,46,51], including climate change, coastal area modifications, and increased nutrient and pollution loads. Recently, Bahía de La Paz has been impacted by tropical storms and hurricanes, leaving numerous boats at the bottom of the sea in coastal areas and nearby islands [52,53]. Some studies have suggested that the abundance of *Gambierdiscus* spp. does not depend on the presence of macroalgae and that certain species are associated with artificial substrates [49,51,54,55,56]. Consequently, the remains of these vessels may represent substrates that promote the potential settlement and proliferation of *Gambierdiscus* and *Fukuyoa* species associated with CFP.

**Figure 12:**
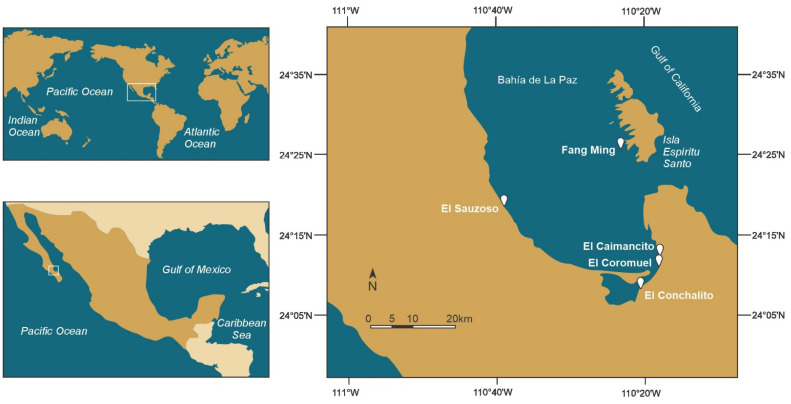
Sampling sites in Bahía de La Paz used to collect *Gambierdiscus* species.

At sampling points near the coast, *G*. cf. *carpenteri* was the dominant species, while the highest diversity was recorded at Fang Ming, a sunken ship converted into an artificial reef near Espiritu Santo Island (Table S2, Figure 12), in addition to *G*. cf. *carpenteri*, *G.* cf. *carolineanus*, and *F.* cf. *yasumotoi* (but not *G.* cf. *caribaeus*). The Gulf of California has 244 islands, islets, and coastal areas [57]. Species of the genera *Gambierdiscus* and *Fukuyoa* are closely associated with the insular areas of the Gulf of California. Morquecho-Escamilla et al. [37] reported the presence of *Gambierdiscus* and *Fukuyoa* on San Jose Island, and Ramos-Santiago et al. [38] reported their presence on La Gaviota Island. Including this study, reports of *Gambierdiscus* and *Fukuyoa* cover only 1.2% of Gulf of California islands, suggesting that these islands are potential habitats for a great diversity of *Gambierdiscus* and *Fukuyoa* species, including novel species.

### 3.2. Biochemical Characterization

#### 3.2.1. Pigments Composition by HPLC-DAD

Pigment analysis has been proposed not only for physiological studies of dinoflagellates [58,59,60,61] but also as a pigment fingerprint for monitoring harmful microalgae species during harmful algal blooms (HABs) [62,63,64,65,66,67]. Using thin-layer chromatography (TLC), Durand and Berkaloff [68] and Indelicato and Watson [69] conducted the first analyses of pigments produced by the genus *Gambierdiscus*, identifying the presence of Chl *a*, Chl *c*1, Chl *c*2, Diadino, Dino, Peri, and ββ-car. Since then, at least ten studies have been conducted to further understand pigment production and composition [63,70,71,72,73] (Table 4). Recently, Malto et al. [27] and Yon et al. [28] described the presence of these compounds using liquid chromatography coupled with mass spectrometry as part of a study on the metabolome of three strains belonging to three species of *Gambierdiscus* isolated from the Philippines and 15 strains of five species from the Atlantic Ocean. The analysis and evaluation of the pigment profile, along with other metabolites (e.g., fatty acids, sterols, carbohydrates, amino acids, and toxins), in various species of dinoflagellates allows for the chemical diversity of secondary metabolites to be explored. Biomarkers can then be identified to differentiate between *Gambierdiscus* species (chemotaxonomy), evaluate the possible presence of ecotypes, and study the metabolism and biosynthesis of toxins. In this study, we detected the presence of seven pigments in *G.* cf. *caribaeus* and *G.* cf. *carpenteri*, and the profiles obtained were compared globally with those from other isolates of species within this genus (Table 4; Figure 6). 

The predominant pigment profile for these two species included Chl *a*, Chl *c*2, Diadino, Dino, Diato, Peri, and ββ-car. Notably, we observed that strain GbSa-7 was distinct from strain GbSa-2; both corresponded to *G.* cf. *caribaeus*, as it produced a higher concentration of Chl *a* (203.79 pg·cell^−1^) and Dino (4.71 pg·cell^−1^). This effect was also observed in the amino acids profile, with histidine (13.04 pg·cell^−1^) detected only in this strain, along with the highest concentration of proline (52.30 pg·cell^−1^; Table 3 and Figure 6). Therefore, we consider that this strain may represent an ecotype. This hypothesis will be further supported by an analysis of the toxin profile and other metabolites (e.g., fatty acids, sterols, and carbohydrates) using analytical methods. 

#### 3.2.2. Amino acid Composition

Analyzing the amino acid profiles of dinoflagellates is useful for exploring the chemical diversity of secondary metabolites; these profiles can serve as biomarkers for species identification in chemotaxonomy. They also facilitate the study of ecotypes, metabolism, and toxin biosynthesis. This analysis also allows for assessments of intracellular changes in response to diurnal variations in light, nitrogen, and nutrients, as well as the physiological status of natural dinoflagellate populations [74,75].

The amino acids profile of *G.* cf. *caribaeus* and *G.* cf. *carpenteri* strains revealed the presence of 14 amino acids: glutamic acid, aspartic acid, serine, glycine, threonine, alanine, arginine, tyrosine, valine, phenylalanine, isoleucine, leucine, lysine, and proline. *Gambierdiscus.* cf. *caribaeus* GbSa-7 was the only strain that presented histidine (13.04 pg·cell^−1^), and it also had the highest concentration of proline (52.30 pg·cell-1; Table 4 and Figure 6). Therefore, we considered that this strain may be a potential ecotype. Figure 6 provides a comparative analysis of the amino acid profile of *Gambierdiscus* detected in this study with those of other dinoflagellate species. 

Studies by Okaichi et al. [76] and Hayashi [77], who used high-speed amino acid analyzers (HSAAA), in addition to the more recent analyses using high-performance liquid chromatography with fluorescence detection (HPLC-FLD) and HPLC-DAD have elucidated the amino acid profiles of various dinoflagellate species from the genera *Prorocentrum*, *Noctiluca*, *Aureodinium*, *Glenodinium*, *Heterocapsa*, *Scrippsiella*, *Alexandrium*, *Gymnodinium*, *Ansanella*, and *Takayama* [74,78]. Recently, Yon et al. [28] also described the presence of these compounds using high-performance liquid chromatography-mass spectrometry (HPLC-MS) as part of the metabolome of 15 strains from five species of *Gambierdiscus* originating from the Atlantic Ocean. Furthermore, for the first time, this study presents a screening of the amino acid profile of *Gambierdiscus* from the Pacific Ocean (Table 5).

### 3.3. Toxicity Assay

CTX-like and MTX-like activity was evaluated using four different assays: *Artemia* assay (ARTOX), mouse bioassay (MBA), marine fish assay (MFA) and fluorescent receptor binding assay (*f*RBA). Toxicity related to CTX-like and MTX-like activity was confirmed in all five clones, although the effects varied across the different toxicological assays.

#### 3.3.1. Mouse Bioassay (MBA)

CTXs-like toxicity was evident in the MBA (18.57 × 10^−4^ µg·kg^−1^·1000 cell^−1^ and 17.08 × 10^−4^ µg·kg^−1^·1000 cell^−1^ in *G.* cf. *caribaeus* GbSa-2 and GbSa-7, respectively, and 29.07 × 10^−4^ µg·kg^−1^·1000 cell^−1^, 8.46 × 10^−4^ µg·kg^−1^·1000 cell^−1^, and 12.54 × 10^−4^ µg·kg^−1^·1000 cell^−1^ in *G*. cf. *carpenteri* GbSa-4, GbSa-5, and GbSa-6, respectively). Clinical signs were also observed for MTX-like activity; however, lethality was only associated with CTX-like activity. The predominant clinical signs were neurological (50–62%), followed by others (10–22%), cardiovascular (7–22%), and gastric (8–16%) across all analyzed strains. In the case of MTX-like activity, neurotoxicity signs predominated (42–70%), followed by gastric (10–25%), others (8–25%), and cardiovascular (8–14%) (Figure 8). These clinical signs were consistent with those described for these types of toxins [79,80]. When comparing the effects of these two types of toxins detected in strains of *G.* cf. *caribaeus* and *G.* cf. *carpenteri*, a higher percentage of neurotoxic clinical signs was observed for MTX-like activity, although it was not lethal in this assay. 

#### 3.3.2. Artemia Assay (ARTOX) 

In ARTOX, toxicity was also higher for CTX-like activity compared to that of MTX-like activity (Figure 9A,B). The highest mortality was recorded at 18 h of exposure to the CTXs-like activity of *G.* cf. *caribaeus* (GbSa-2: 45 ± 3% and GbSa-7: 38 ± 2%) compared to those of the three strains of *G.* cf. *carpenteri* (GbSa-4: 38 ± 1%; GbSa-5: 90 ± 6%; GbSa-6: 78 ± 5%). Clinical signs in adult brine shrimp exposed to CTX-like activity were characterized by changes in swimming behavior, starting at 6 h in *G.* cf. *caribaeus* and *G.* cf. *carpenteri*. These changes included loss of swimming orientation characterized by circular swimming and rising and falling from the surface to the bottom, which intensified between 6 and 24 h, with gasping, lethargy, and paralysis of the thoracic appendages. These clinical signs were consistent with those described for this type of toxin in fish extracts and *Gambierdiscus* and *Fukuyoa* extracts [81,82]. Although mortality associated with MTX-like activity was lower, several clinical signs were observed, such as erratic swimming, lethargy, head-up posture, under-reactive response to stimuli, and gasping within the first 12 h. By the end of the exposure, 80% of the brine shrimp had recovered. The toxic effects of some species of benthic dinoflagellate species, such as *Prorocentrum*, *Ostreopsis*, *Gambierdiscus*, and *Fukuyoa*, have been previously demonstrated by Neves et al. [83], Heredia-Tapia et al. [84], Ajuzie [85], Faimali et al. [86], and Gong et al. [87]. Additionally, *Gambierdiscus* and *Fukuyoa* have been used as CTXs-like and MTXs-like bio-vehicles for metabolic studies of these polyether toxins in marine fish [88,89].

#### 3.3.3. Marine Fish Assay

The MFA elucidated an opposite effect to that of the MBA, as MTX-like activity was highly ichthyotoxic for *S. rivoliana* larvae. The toxicological effects of CTXs-like activity produced by *Gambierdiscus* in fish (larvae, juveniles, and adults) have been evaluated under controlled conditions in various freshwater and marine species using different methods, such as feeding with contaminated fish pellets, dinoflagellates that produce these toxins, or a bio-vehicle like brine shrimp, as well as injections, and in some cases, dissolved toxins in seawater [89,90,91,92]. Lewis [93] described CTXs as potent ichthyotoxins. Pure ciguatoxin-1 (CTX-1) and CTX-2 added to water containing *Gambusia affinis* induced similar clinical signs, including pronounced opercular movement and uncoordinated swimming, preceding death.

Previous studies have reported the negative effects of CTXs-like activity (ranging from behavioral changes to toxicogenomic effects) on fish embryos, larvae, juveniles, and adults of marine and freshwater species (e.g., *Thalassoma bifasciatum*, *Epinephelus fulvus*, *Lutjanus apodus*, *L. mahogoni*, *Micropterus salmoiedes*, *Oryzias melastigma*, *Oryzias latipes*, *Gambusia affinis*, *Serranus cabrilla*, and *Pterois volitans*). These effects include skin color variations, hypoactivity, loss of equilibrium, erratic swimming, jerky feeding movements, loss of orientation, and death, as well as induced hyperkinetic twitching, severe spinal deformities, and an inability to feed [90,91,92,93,94,95,96,97,98,99,100,101,102,103,104,105,106]. 

Feeding cichlid fishes (*Oreochromis niloticus* and *O. mossambicus*) with *Gambierdiscus* causes balance abnormalities and erratic spiral swimming behavior [99]. Other studies under controlled laboratory conditions have also shown that feeding with different concentrations of *Gambierdiscus* causes changes in coloration, inactivity, adverse effects on swimming and orientation, an inability to eat, and, in some cases, death in reef fish species such as *Thalassoma bifasciatum*, *Acanthurus bahianus*, and *A. chirurgis* [91,92,94,98]. *Gambierdiscus* also causes color modifications and behavioral changes in *Chromis chromis* and a decrease in liver size and coloration of this organ in *Serranus cabrilla* [101]. Purified extracts of toxins from this dinoflagellate caused changes in Na^+^ content in the muscle of *Chelon labrosus* [95], epithelial degeneration of the intestine, dilation of the gill filaments in *Pomacentrus wardi* and *Chromis nitida* [96], and mortality in *Gambusia affinis* [93]. Galarza et al. [107] described the effect of CTXs (obtained from barracuda) on the aggregation of pigment granules in the melanophores of teleost fish. A detailed pathological study using transmission electron microscopy [101] demonstrated structural alterations in the liver of the cabrilla *Serranus cabrilla* related to the ingestion of *Gambierdiscus* in the diet. Marked intercellular fibrosis, a decrease in the number and length of microvilli in the sinusoidal and canalicular areas of the hepatocytes, lipid accumulation, a high number of binucleated cells, nucleolar disorganization, loss of condensed heterochromatin in the periphery of the nucleus, nuclear pyknosis, dilated rough endoplasmic reticulum, and dilated mitochondria were observed.

Although MTXs-like toxins are among the most potent ichthyotoxins [108] and some of the largest and most powerful toxins known [109,110], their accumulation in fish and their ichthyotoxicity has not been extensively evaluated [108,111,112]. MTX was first found in the viscera of the surgeon fish *Ctenochaeus straiatus*, which bears the Tahitian name “maito” and has been occasionally associated with CFP [110]. The amphiphilic nature of MTX, derived from the uneven distribution of polar functional groups in the molecule, is thought to play an important role in its interaction with cell membranes, contributing to its potent activity [108,110,113,114].

MTXs, which are also produced by *Gambierdiscus*, have been described for their negative effects on the gills of the freshwater white cloud mountain fish *Tanichthys albonubes*. MTX caused marked vacuolization of the cytoplasm of chloride cells in the epithelium. Additionally, marked apoptosis was observed not only in the gill epithelial cells but also in all mesenchymal cells of the gill filaments, except in the cartilage in the terminal state [115].

Igarashi et al. [108] evaluated the ichthyotoxic activity of MTX added to water in *T. albonubes*. The LC_50_ of MTX in these fishes in Ca^2+^-free media (pH 8) was 5 nM, but this value decreased significantly to 3 pMa after the pH increased to 8 and the Ca^2+^ concentration increased to 2 mM. Therefore, the authors suggested that in a marine environment, MTX is 2000 times more toxic to fish than brevetoxin-3 (PbTx-3), highlighting the need for special attention to be paid to the extraordinarily high ichthyotoxicity of MTX.

Kholi et al. [111] described the accumulation of MTXs under controlled conditions in carnivorous marine fish, using *Gambierdiscus* as a source of these toxins. Juvenile mullet (*Aldrichetta forsteri*) were fed pellets containing *G. australes*; these were also used as food for the snapper *Pagrus auratus*. The transfer of MTX-1 was confirmed by LC-MS/MS detection in viscera, liver, and muscle samples.

In this study, *S. rivoliana* larvae exposed to dissolved MTX-like activity presented clinical signs after 6 h after exposure. MTX-like activity was highly ichthyotoxic for *S. rivoliana* larvae, which exhibited changes such as abnormal horizontal position, loss of balance, loss of buoyancy, control leading to sinking to the bottom, abnormal vertical orientation with a head-down posture, behavioral changes in swimming, constant pulses of hypoactivity and hyperactivity, erratic movements, shaking, body arching, abnormal respiration activity, hyperventilation, irregular ventilation, head shaking, and notable skin darkening before death. After 24 h of exposure, the maximum mortality of *S. rivoliana* larvae was 60 ± 4% in those exposed to *G.* cf. *caribaeus* (GbSa-2 and GbSa-7) and *G.* cf. *carpenteri* (GbSa-4). The lowest mortality (20 ± 1%) was recorded in the groups of larvae exposed to MTX-like activity from *G.* cf. *carpenteri* (GbSa-5 and GbSa-6) (Figure 9B). However, after 24 h of the acute test, the surviving larvae were moribund, with most of them found at the bottom.

During epizootics and mass mortality events of reef fish (*Pomacanthus paru*, *P. arcuatus*, *Holocanthus tricolor*, *Centropyge argi*, *Scarus taeniopterus*, *Chromis cyaneus*, *Diodon holocanthus*, *Cantherhines macroceros*, *Acanthurus chirurgis*, *Chaetodon sedentarius*, and *C. capistratus*) observed in Florida and the Caribbean, Ladsberg [116] suggested a possible association with benthic HABs caused by dinoflagellates of the genera *Gambierdiscus*, *Prorocentrum*, and *Ostreopsis* and cyanobacteria of the genus *Lyngbya* as potential etiologies of these events. In Cabo Pulmo in the Gulf of California, Nuñez-Vazquez et al. [34] described epizootics and mass mortality of reef fish (tons) potentially associated with benthic HABs, highlighting the presence of *Gambierdiscus*, *Ostreopsis*, *Prorocentrum*, and *Lyngbya* species. Recently, massive die-offs of reef fish in Florida have been described, with individuals showing signs of acute poisoning similar to those observed in some trials of acute exposure to toxins from *Gambierdiscus* and other benthic dinoflagellates. Therefore, it is important to study whether *Gambierdiscus* toxins are associated with these epizootics, as has been recently suggested.

Based on existing knowledge and the results observed in the present study, new studies should be conducted that evaluate the ecotoxicological role of toxins and other related compounds from *Gambierdiscus* and other benthic species in the health of fish during different life stages in the wild and under aquaculture conditions. In the latter case, their importance as future animal models to study the metabolism and biosynthesis of CFP toxins and other syndromes of benthic origin should also be explored.

### 3.4. Fluorescent Receptor Binding Assay (fRBA) 

Although fRBA is not specific in detecting the presence of toxins, the sensitivity of this test has demonstrated its efficiency in fish tissues, *Gambierdiscus,* and *Fukuyoa* for detecting compounds related to VGSC site 5, such as CTX and PbTx [42,117,118]. In this study, *G.* cf. *caribaeus* showed concentrations based on the VGSC competitor of 2.19–2.59 ± 0.24 pg eq PbTx cell^−1^, a medium-to-low concentration similar to that reported for *G. caribeaus* from Cuba [42]. The estimated concentration for *G.* cf. *carpenteri* samples ranged between 1.15–2.51 ± 0.31 pg eq PbTX·cell^−1^, which is three times lower than that reported by Vacarizas et al. [45] (7.48 ± 0.49 pg PbTx eq·cell^−1^). Although PbTx was used as a standard reference to quantify the activity of toxins related to the sodium channel, previous studies have reported better sensitivity during quantification when using CTX competition as a reference standard [41,117] compared to standardized tests such as CBA-N2a and ELA [47,118]. In the present study, strains of *G.* cf. *caribaeus* and *G.* cf. *carpenteri* (Table 6) support the conclusion that these species present medium-to-low toxicity compared to *G. exentricus* and *G. polynesiensis*, which are representatives of the genus with higher CTXs production per cell. Future studies should compare the binding percentage with a CTX standard due to variations in the capacity to bind VGSC, as the extracts tested are likely to contain a mixture of analogs that could affect the sensitivity and binding signal, which could differ when quantifying using the brevetoxin standard.

Table 6 describes and compares the main toxicity assays performed at the global level and the marine toxins detected in the *G. caribaeus* and *G. carpenteri* strains. Various assays and analytical techniques have been used to determine the toxicity and chemical composition of the toxins produced by these two species in different parts of the world. Toxicity varies from nontoxic strains to those that present high toxicity. Some reports indicate 1.4 ± 0.6 fg CTX3C eq cell^−1^ in strains from Hawaii [126]), 0.3–1.4 fg CTX3C eq cell^−1^ in strains isolated from the Caribbean [39], and 7.48 ± 0.49 pg PbTx-3 eq cell^−1^ and 12.36 ± 4.38 pg PbTx-3 eq cell^−1^ in strains from the Philippines (all *G. carpenteri*) [27,44,45]. In addition, *G. carpenteri* strains from Australia have been reported to have a toxicity of 2.4 mg kg^−1^ per MBA, and Ramos-Santiago et al. [38] reported a CTX-like toxicity of 5.9 mg kg^−1^ by MBA in Isla Gaviota, Gulf of California.

In *G. caribaeus*, a toxicity of 0.66 ± 0.34 fg CTX3C eq cell^−1^ was described in strains from the Caribbean [42], 1.0–1.6 fg CTX3C eq cell^−1^ in strains from Hawaii [118,126], 15.89 fg CTX1B eq cell^−1^ in strains from the Canary Islands (127), 0.54 fg CTX3C eq cell^−1^ in strains from the Gulf of China [123], and 0.6 ± 0.2 pg cell^−1^ and 9.8 ± 0.6 pg cell^−1^ (Gambierone) and 7.1 ± 4.2 pg cell^−1^ (44-methylgambierone) in strains from St. Thomas, US Virgin Islands [24]. For the strains of *G. caribaeus* and *G. silvae*, a new ciguatoxin was isolated in the Caribbean and named C-CTX-5 [24,25]. This finding represents a significant advance in research on the origin of CFP in the Caribbean, as the precursor toxin from its algal source and its relationship with CTXs in fish that cause the disease was previously unknown, hindering the development of detection methods, diagnostics, and monitoring programs. In the same study, various in vitro and chemical techniques were applied to identify the new analog (C-CTX-5), and it was verified that the treatment of this analog using fish liver microsomes converted it to C-CTX1/2, the dominant C-CTX in ciguatoxic fish from the Caribbean. Recently, C-CTX-5 was also identified for the first time in an “amberjack” (*Seriola* sp.) involved in a CFP case in the Canary Islands, Spain [127]. Based on this, it is necessary to investigate whether *G. caribaeus* strains from other latitudes, such as those in the present study, can also produce this new ciguatoxin. 

The genetic identification of the *Gambierdiscus* and *Fukuyoa* species evaluated in this study and chemical analysis of the toxins will be carried out in a subsequent study using analytical tools, given the complexity of obtaining standards and other inputs necessary to detect and analyze CTXs, MTXs, and other related compounds. The toxicity and composition of CTXs, MTXs, and other related compounds in these two species are also described in Table 6.

Over the past decades, the occurrence and geographic distribution of CFP have notably expanded due to anthropogenic activities and climate change, leading to an increase in human illness, greater public health impacts, and larger economic losses. The global spread of CFP has made *Gambierdiscus* and its toxins a concern for environmental and human health worldwide [22].

In the PBC, cases of CFP are rare and sporadic. However, at least 240 human cases have been recorded in the southern region due to the consumption of the meat, liver, and heads of carnivorous fish, such as snappers (*Lutjanus* spp.), cabrillas, and groupers (*Epinephelus* and *Mycteroperca*), caught near islands and islets [11,12,13,14,15,16,17]. Additionally, epizootics and the massive mortality of reef fish potentially associated with benthic HABs have been reported, with *Gambierdiscus*, *Ostreopsis*, *Prorocentrum*, and *Lyngbya* species being primarily detected [34].

The PBC is also a region of notable fishery importance, supporting artisanal fisheries that exploit numerous species of teleost fish, elasmobranchs, mollusks, and crustaceans. In the Gulf of California, coastal fisheries target around 70 species, with an annual catch of nearly 200,000 tons. Sport fishing is also prominent, targeting species such as yellowfin tuna (*Thunnus albacares*), skipjack (*Katsuwonus pelamis*), billfish striped marlin, blue marlin (*Makaira nigricans*), black marlin (*Makaira indica*), sailfish (*Istiophorus platypterus*), swordfish (*Xiphias gladius*), dorado (*Coryphaena hippurus*), yellowtail amberjack (*Seriola lalandi*), longfin yellowtail (*Seriola rivoliana*), snappers (*Lutjanus* spp.), “groupers” (*Ephinpehelus* spp. and *Mycteroperca* spp.), and around 40 species of sharks, including *Mustelus*, *Carcharhinus*, *Alopias*, *Sphyrna*, and *Squatina* [128]. The region is also important for activities such as whale watching (e.g., *Eschrichtius robustus*, *Megaptera novaeangliae*, *Balaenoptera musculus*, and *Balaenoptera physalus*) and observing dolphins (e.g., *Tursiops truncatus* and *Delphinus* spp.), sea lions (*Zalophus californianus*), sea turtles, whale sharks (*Rhincodon typus*), reef fish, a great diversity of invertebrates, and algae [128,129,130]. In recent decades, the southern PBC has experienced increased tourism and urban growth, increased demand for services, and the development of marine fish farming, bivalve aquaculture, and shrimp production in Bahía de La Paz.

The annual economic contribution of sport fishing in the southern PBC was estimated to be $80,801,119 USD between 2008 and 2010, equivalent to 1.4% of the Gross Domestic Product (GDP) of Mexico during that period, according to the National Institute of Geography and Statistics (INEGI) [131,132].

In this study, in addition to *G. caribaeus* and *G. carpenteri*, we report the presence *Gambierdiscus* (*G.* cf. *carolinianus* and *G.* cf. *toxicus*) and *Fukuyoa* (*Fukuyoa* cf. *yasumotoi*) species in the PBC, where cases of CFP have occurred. The Gulf of California is a potential hotspot for the biodiversity and endemism of benthic dinoflagellate species, which provides an important opportunity to research CTXs, MTXs, CFP-related compounds, other polyether toxins, and secondary metabolites. Studies must be conducted that aim to monitor and evaluate the toxicity, toxin composition, and secondary metabolites of different species of *Gambierdiscus* and *Fukuyoa* in this region. The results of these studies are essential for protecting human and animal health. These studies also represent an opportunity to advance research on chemical diversity (metabolomics), which could contribute to characterizing the species and ecotypes present and their potential biotechnological or therapeutic applications.

## 4. Materials and Methods

### 4.1. Isolated and Algal Cultures

During August 2022, samples of the macroalgae *Dyctiota* spp. and *Padina* spp. (associated with corals) were collected by snorkeling at a depth of less than 4 m in the coral reef of El Sauzoso, from artificial substrates at Playa Coromuel, from the rocky reef at El Caimancito, and from the sandy intertidal plane of El Conchalito. Additional samples were collected at the Fang Ming scuba diving site in Espiritu Santo Island (Figure 12).

Samples were carefully incubated in Nalgene 1-L bottles and immediately transported to the Marine Toxins and Amino Acids laboratory of CIBNOR. *Gambierdiscus* cells were isolated individually from macroalgae, rocks, and sandy sediments using a capillary micropipette under an inverted microscope (Labomed, TCM 400, Hicksville, NY, USA). To establish the culture, the isolated cells were transferred into 24-well flat bottom plates (Costar, Boston, MA, USA) with 1 mL of modified K medium [133] containing 1 mg/L of germanium oxide. After 45 days, the wells containing more than 20 cells were transferred to flat bottom culture tubes (Pyrex®, Sunderland, UK) containing 20 mL of medium. These cultures were maintained in the culture room of the CIBNOR microalgae collection (25 ± 2 °C, 35 salinity, a 12:12 light-dark cycle, and irradiance of 35 mmol photon m^−2^ s^−1^ supplied by LED light (MAGGA T8 LED STD 18 W, CDMX, Mexico)). All cultures were carried out under these conditions.

Growth rates and generations per day were calculated according to the methods described by Guillard and Ryther [134] and Guillard [135]. Briefly, triplicate cultures of each species were maintained in stock cultures in 300-mL Erlenmeyer flasks with 100 mL of medium at an initial density of 50 cells·mL^−1^. Every seven days, 2-mL samples were fixed with Lugol’s solution Utermöhl for cell counts on Sedgwick-Rafter slides under a compound light microscope (Olympus BX41, Tokyo, Japan). Serial culture batches were established in 2.8-L Fernbach flasks (Pyrex®) with 1 L of culture medium to increase biomass to evaluate morphological taxonomy, pigments, and amino acids, as well as the mouse bioassay, fluorescent receptor binding assay (fRBA), artemia assay (ARTOX), and larvae fish assay.

### 4.2. Morphological Identification

#### 4.2.1. Light Microscopy LM

Observations, measurements, and photomicrographs were conducted on 30 randomly selected live *Gambierdiscus* cells. Cell size, including depth (ventral/dorsal distance), width (transdiameter), and length (apical/antapical axis), was recorded under a microscope (Olympus BX41, Japan) equipped with a camera (SC-MOUNT-YW500 Sony sensor IMX335, ImageView software 2.0), and the system was calibrated using a micrometric slide (1–1000 µm) with achromatic (40×/0.65 Ph2) and 100×/1.25 Oil Ph3 ∞/−) Olympus objectives. Ten milliliters of the culture were collected in 15-mL conical tubes, and cells were fixed with 4% glutaraldehyde. To identify cell plates, a 5-mL aliquot was centrifuged at 1200× *g* for 2 min, the fixative was removed, and cells were washed three times with distilled water before being fixed with 4% sodium hypochlorite to dissociated cell plates. Plate tabulation nomenclature followed the description of Ramos-Santiago et al. [38]. Another 2 mL of fixed sample was washed again with distilled water by centrifugation and treated with Fluorescent Bright 28 (Sigma Aldrich, St. Lois, MO, USA) [136]. Fluorescent Bright 28 (blue emission) and natural autofluorescence of chloroplasts were documented using a scanning laser confocal microscope (Leica^®^ DMi8, Wetzlar, Germany).

#### 4.2.2. Scanning Electron Microscopy SEM

Ten milliliters of culture were fixed with osmium in 15-mL sterile conical tubes (Corning®, Corning, NY, USA). After 24 h, the cell pellet was filtered through membrane filters with a 0.2-µm pore size and 25-mm diameter (Gelman Science, Bloomfield Hills, MI, USA). Cells were washed four times with filtered distilled water and sequentially dehydrated with 30, 50, 75, 95, and 100% EtOH. After critical-point-drying, the cells were transferred and mounted on 12-mm carbon tabs PK/10 (West Chester, PA, USA), coated with gold, and examined under a scanning electron microscope (Hitachi^®^ S300N, Tokyo, Japan) using image capture software (Quartz PCI 200).

### 4.3. Biochemical Characterization

#### 4.3.1. High-Performance Liquid Chromatography (HPLC)

The pigment composition of five strains of two species of *Gambierdiscus* species was analyzed by HPLC-UV/VIS according to the methods [66,137], with some modifications. Briefly, 30 mL of *Gambierdiscus* cells were collected on a glass microfiber filter GF/F (Ahlstrom—Munksjö, Sweden). The filters were extracted with 2 mL of 100% acetone in an ice bath, hand-ground with a glass rod, and stored overnight at −40 °C for full extraction. The extract was recovered after centrifugation (3000× *g* for 5 min). A 200-µL aliquot of the extract was mixed with 100 µL of 0.5 N ammonium acetate and injected through a 100-µL loop into an HPLC system (Hewlett Packard series 1260) equipped with a photodiode array detector (DAD, 1.2 nm optical resolution). Pigments were separated and quantified using an isocratic HPLC in reverse-phase mode, as described by Vidussi et al. [137] and Bustillos et al. [66]. The mobile phase consisted of MeOH (0.5 N aqueous ammonium acetate, 70:30% *v*/*v* (solvent A), and MeOH [solvent B]), with the following gradient (minute (percent of solvent A-percent of solvent B)): 0 (75–25), 1 (50–50), 15 (0–100), and 19 (75–25). Quantification was based on the absorbance at 440 nm, and the response factor (peak area/pigment concentration) for each pigment was determined as described by Mantoura and Repeta [138]. Pigment identification was based on retention time, spectral characteristics, and chromatography using certified commercial standards (International Agency for 14C determinations, Hørsholm, Denmark).

#### 4.3.2. High-Performance Liquid Chromatography-Diode Array Detection (HPLC-DAD) for Amino Acid Composition

The amino acid composition of five isolates of two species of *Gambierdiscus* was analyzed by HPLC-DAD according to Gratzfeld-Huesgen [139] and AOAC [140], with some modifications. A 150-mL volume of *Gambierdiscus* cultures was collected on a GF/F filter (Sartoriuos, Goettingen, Germany), and the filter was dried in an oven (Symphony™ VWR^®^, Radnor, PA, USA) at 60 °C. Dried samples were hydrolyzed in a mixture of 6 N hydrochloric acids/phenol at 110 °C for 24 h. After acid hydrolysis, samples were washed with deionized water and dried in a rotary evaporator (IKA^®^-WERKE RV05, Staufen im Breisgau, Germany) before undergoing oxidation with performic acid and alkaline hydrolysis with 4.2 M sodium hydroxide (NaOH). Finally, samples were filtered through syringe filters with 0.2–0.45 µm pore sizes before analysis on the HPLC system. An L-amino acid kit from SIGMA Chemicals Corp. (Saint Louis, MO, USA) was used. O-phthalaldehyde (OPA) solution: o-phthalaldehyde and 3-mercaptopropionic acid (Agilent Technologies Part no. 5061-3335, Santa Clara, CA, USA); 9-fluorenylmethylchloformate (FMOC) solution: 9-fluorenylmethylchloformate (Agilent Technologies Part No. 5061-3337) and Borate Buffer 0.4 M at PH 10.2 (Part Number: 5061-3339). For calibration, the following profile of L-amino acids (SIGMA Chemicals Corp., Saint Louis, MO, USA) was used: aspartic acid (Asp), glutamic acid (Glu), serine (Ser), histidine (His), glycine (Gly), threonine (Thr), alanine (Ala), arginine (Arg), tyrosine (Tyr), valine (Val), phenylalanine (Phe), Isoleucine (Iso) leucine (Leu), lysine (Lys), and Proline (Prol) at a concentration of 0.5 µmol/mL in 0.1 N HCl.

Sample derivatization method: derivatization with OPA was used for primary amino acids and FMOC for secondary amino acids. Automatic mixing of 1 µL of the sample was performed in the injector, with 5 µL borate buffer at pH 10.4, 1 µL OPA, and 1 µL FMOC. An Agilent Technologies 1100 series HPLC with a UV-VIS diode array detector was used. A Hypersil 5 AA-ODS (Agilent Technologies) reversed-phase column (200 mm length, 2.1 mm diameter, and 5 μm particle size [Part No. 79916AA-572]) was used. A C18 4 × 3.0 mm Phenomenex column guard (Part No. AJ0-4287) was used to protect the column. Detection was performed by UV at 338 nm for primary amino acids and at 262 nm for secondary amino acids at 40 °C with a flow rate of 0.450 mL/min and an injection volume of 8 µL using a binary gradient program. The mobile phase was prepared based on a binary gradient (A, B), with phase A of 20 mM sodium acetate buffer with 0.018% (*v*/*v*) triethylamine and 0.03% tetrahydrofuran, and phase B consisting of 20% of 100 mM sodium acetate buffer, 40% acetonitrile, and 40% MeOH.

### 4.4. Toxicity Assay

#### 4.4.1. Toxins Extraction

Batch cultures were harvested during the early exponential phase, and the number of cells was recorded. Harvesting was carried out by concentrating cells on glass microfiber filters with a pore size of 0.7 μm and a diameter of 47 mm (Whatman™, Maidstone, UK). Toxin extraction from *Gambierdiscus* cells was performed following the method described by Chinain et al. [141]. Methanol (50 mL) was added, and the filters were macerated with a glass rod for 5 min, followed by sonication for 20 min (Branson^®^ 1210, Branson, MO, USA) three times. Methanolic extracts were dried at 40 °C using a Bücher Rotary evaporator. The extract was resuspended and partitioned in a separating funnel by adding dichloromethane (DCM) (50 mL per million cells) and MeOH:H2O (6:4. *v*/*v*) (25 mL per million cells) [142,143]. Two fractions were obtained: the methanol:water phase containing MTX and the DCM fraction containing CTX. 

#### 4.4.2. Mouse Bioassay (MBA)

The toxicity of *Gambierdiscus* was assessed using MBA for CTX-like and MTX-like toxins. The mouse model was maintained under comfortable conditions (temperature of 22 ± 1 °C, a 12:12 light:dark cycle, and a maximum humidity of 60%) in compliance with the Official Mexican Standard NOM-062-ZOO-1999. The mice were provided with bedding made of Aspen shavings from Nepco and were fed sterile formulated pellets (Zeigler, Gardners, PA, USA) and sterile acidified water provided *ad libitum*. The DCM and MeOH:H_2_O phases were evaporated and resuspended in 1% Tween 60 and 0.9% sterile saline solution. A 1 mL dose was intraperitoneally (i.p.) injected into male mice weighing 18–20 g each (strain CD-1 Harlan Laboratories, CDMX, Mexico) divided in groups of two animals, with a control group injected with Tween solution. Biological activity and toxicity were determined following the recommendations described by Satake et al. [79,142,143]. The mouse unit (MU), defined as the i.p. The LD_50_ dose for a 20-g mouse was calculated according to Lewis [77]. To determine the toxicity of a mixture of ciguatoxins, the following formula was used:

(1) $$log\;MU=2.3log\left(1+\frac{1}{T}\right)$$

To determine the toxicity of a mixture of maitotoxins, the following formula was used:

(2) $$log\;MU=6.7log\left(1+\frac{1}{T}\right)$$

Continuous monitoring was conducted every hour for 24 h, during which the clinical signs of poisoning in mice were recorded and reported as a percentage according to the affected body system. 

#### 4.4.3. *Artemia salina* Assay (ARTOX) 

The *A. salina* toxicity assay was performed to evaluate the acute toxicity of the CTXs-like and MTX-like extracts, following the procedure described by Heredia-Tapia et al. and Neves et al. [82,83]. Adult brine shrimp (*A. salina*) were cultured in plastic containers and exposed to the extracts (1 mL in 1% Tween 60 and 0. 9% sterile saline solution) of *G.* cf. *caribeaus* and *G.* cf. *carpenteri*, equivalent to 450,000 cel mL^−1^ in a glass container of brine shrimp (n = 10). Clinical signs were observed over a period of 24 h, with observation at 6, 12, 18, and 24 h, to determine the mortality rate associated with each extract.

#### 4.4.4. Marine Fish Assay

Groups of *S. rivoliana* larvae were conditioned for 24 h in cylindrical glass tanks containing 400 mL of seawater with aeration at 25 °C, after which the water was changed. To evaluate the acute toxicity of the extracts, 3 mL of CTXs-like and MTXs-like extracts, equivalent to 450,000 cel mL^−1^ of *G*. cf. *caribaeus* and *G*. cf. *carpenteri*, were added to 0. 9% sterile saline solution. Two control groups were established, one with saline solution and the other with seawater. Mortality was recorded for each treatment at 6, 12, 18, and 24 h, and clinical signs were documented following the Organisation for Economic Co-operation and Development OECD guidelines for testing chemicals (Test No. 203: Fish, Acute Toxicity Test). Mortality was reported as a percentage, comparing the number of survivors to dead individuals in the control groups with the number of survivors to dead *S. rivoliana* larvae in the treatments with the dissolved CTXs-like and MTXs-like extracts.

### 4.5. Fluorescent Receptor Binding Assay (fRBA) 

The SeaTox Research Inc. (Wilmington, NC, USA) Brevetoxin/Ciguatoxin RBA test kit was used to detect the presence of compounds with binding affinity to VGSC site 5, which is specific for the receptor but not for toxins (e.g., PbTx-1, PbTx-2, and PbTx-3 and ciguatoxin P-CTX-1, P-CTX-3C, and C-CTX-1) [117]. In this assay, the receptor was mixed with a fluorescent ligand and an unlabeled ligand. In the presence of a low competitor (e.g., ethanol control or 0.1 ng/mL^−10^ log in the standard brevetoxin assay), the receptor site was predominantly bound by the fluorescent ligand, resulting in high fluorescence levels in the samples. When the concentration of a competitor was high (e.g., 1 or 0.1 µg/mL standard brevetoxin; -6 and -7 log in the assay), most receptor sites were occupied by the unlabeled ligands, and low fluorescence levels were measured from the sample. 

The reagents were prepared in 1× buffer (10 mL of buffer from the kit with 90 mL of sterile distilled water) and stored at 4 °C until use. Fluorescent ligands were resuspended in 60 µL of HPLC-grade ethanol to create a 100× solution in an amber glass vial, sonicated, and vortexed to ensure resuspension. A 1× working solution was prepared by diluting 55 µL of fluorescent ligand (100×) in 5.5 mL of assay buffer (1×) and kept at –20 °C until use. The brevetoxin standard (100 µg/mL) was prepared by adding 35 µL of ethanol directly to the vial, followed by sonication and vortexing to achieve a final concentration of 1 µg/mL. A series of five 1:10 dilutions (0.1–0.00001 µg/mL standard brevetoxin) were prepared in HPLC vials, starting with 10 µL of brevetoxin standard stock 1, adding 90 µL of solvent for standard 2, and continuing this process to standard 5. The samples were stored at −20 °C until use. Synaptosome receptors were refrigerated until use and were completely and gently resuspended in 5 mL of sterile distillate water. During the test, the resuspended aliquot was always kept on ice. Samples were prepared by evaporating a 3-mL aliquot at a concentration of 400,000 cell·mL^−1^ for fRBA, following the procedure described by Darius et al. [118]. The *Gambierdiscus* DCM phase, corresponding to the ciguatoxin mixture, was resuspended in 1× assay buffer. An extract of *Karenia brevis* with a known profile (PbTx-2 and PbTx-3 by LC-MS/MS) was tested, and ethanol was used as a control.

The fRBA reaction assay plate was carried out in qualitative mode: single concentration (triplicate), 5 µL added to each series of the standard dilution, 5 µL of *Gambierdiscus* extract, 5 µL of *K. brevis* extract, and 6 wells with 5 µL of ethanol control. Then, 195 µL of assay buffer and 50 µL of cold synaptosome solution were added to each well, and the plate was incubated and covered on ice for 30 min with agitation and in darkness. Afterwards, 50 µL of fluorescent ligand (1×) working solution and 200 µL of assay buffer (1×) were added to each well and incubated in darkness for 60 min on ice, with gentle mixing on a table shaker. During incubation, 250 µL of 1× assay buffer was passed through the wells of the filter plate provided in the kit assay and dried using a vacuum system until no liquid remained on the paper towel. After incubation, the reaction mixtures were transferred from the plate to the filter plate and dried in a vacuum manifold system. The filter wells were rinsed with 500 µL of assay buffer, and air was removed from the plate by vacuum for at least 30 s after the final rinse. The plate filter was read at 490–10 nm (excitation filter) and 520–10 nm (emission filter), with wavelengths determined from the extra fluorescent ligand provided and the empty filter plate to minimize background fluorescence, using a fluorescent plate reader (Thermo Scientific Varioskan Flash 3001-2158, Waltham, MA, USA). 

Data were analyzed as the relative fluorescence unit (RFU) of total binding (%), using an ethanol control (100% fluorescent ligand). Background RFU values were subtracted from the final RFU data for each well, and the values were extrapolated to picograms equivalent brevetoxin per cell (pg eq PbTX) from the standard curve generated by the brevetoxin standard. 

## 5. Conclusions

1. *Gambierdiscus* cf. *carpenteri* is the most abundant species in Bahía de La Paz and is associated with the production of toxins related to CFP. The presence of G. cf. *caribeaus, G.* cf. *carolineanus*, *G.* cf. *toxicus*, and *F.* cf. *yasumotoi* was also recorded.
2. There are differences in pigment production and in the amino acid profiles between the strains *G.* cf. *caribaeus* and *G.* cf. *carpenteri*. These results suggest the presence of ecotypes, even among strains of the same species. The biochemical profiles could potentially establish chemotaxonomic criteria for the identification of *Gambierdiscus* species.
3. Toxicity for CTX-like and MTX-like activity was confirmed in all strains of *G.* cf. *caribeaus* and *G.* cf. *carpenteri*. CTX-like and MTX-like toxicity produced a positive result in the MBA and caused the mortality of *A. salina*. In the marine fish assay, the CTXs-like extracts presented a low-to-medium toxicity through competition for VGSC site 5, as quantified by the fluorescent receptor binding assay.
4. The differences in the responses across the various assays suggest that *G.* cf. *caribeaus* and *G.* cf. *carpenteri* from Bahía de La Paz may produce different metabolites associated with the genus *Gambierdiscus*.
5. The Gulf of California is a potential hotspot of biodiversity and endemism for benthic dinoflagellate species and a key area for research on CTXs and MTXs, CFP-related compounds, other polyether toxins, and secondary metabolites.

## Figures and Tables

**Table 1:** Morphological measurements of *Gambierdiscus* strains from El Sauzoso, Bahía de La Paz, obtained through light microscopy.

	Cell Size (µm)			Apical Pore Plate Po (µm)			Po Apical Pore (µm)			1p Plate (µm)	
Specie/Strain	D	W	D/W	L	W	L/W	Number	Diameter	L	W	L/W
*G.* cf. *caribaeus*/ GbSa-2	87.15 ± 4.73	83.32 ± 4.90	1.05 ± 0.56	10.04 ± 0.35	6.21 ± 0.31	1.62 ± 0.07	37.16 ± 2.32	0.48 ± 0.05	49.88 ± 4.24	37.55 ± 3.09	1.29 ± 0.13
*G.* cf. *caribaeus*/ GbSa-7	79.32 ± 8.75	70.29 ± 5.98	1.13 ± 0.63	9.91 ± 1.22	6.23 ± 0.89	1.59 ± 1.38	37.50 ± 5.23	0.41 ± 0.05	45.55 ± 3.31	36.19 ± 1.99	1.28 ± 0.10
*G.* cf. *carpenteri*/ GbSa-4	77.67 ± 7.12	71.31 ± 5.74	1.09 ± 1.24	10.35 ± 0.49	6.17 ± 0.37	1.68 ± 1.32	40.50 ± 3.24	0.41 ± 0.09	55.59 ± 2.95	35.99 ± 3.72	1.62 ± 0.19
*G.* cf. *carpenteri*/ GbSa-5	79.44 ± 6.73	71.32 ± 6.55	1.11 ± 1.03	11.03± 0.95	7.28 ± 1.71	1.52 ± 0.56	41.50 ± 1.58	0.42 ± 0.10	46.99 ± 2.26	30.90 ± 5.23	1.55 ± 0.29
*G.* cf. *carpenteri*/ GbSa-6	79.59 ± 8.63	71.34 ± 9.25	1.12 ± 0.93	10.47± 1.49	6.67 ± 1.29	1.57 ± 1.16	40.00 ± 2.49	0.42 ± 0.07	47.61 ± 4.33	29.86 ± 4.15	1.61 ± 0.24

Depth (D), Width (W), Length (L), and ratio for measurement plate (L/W). Data expressed as means ± standard deviation. (n = 30).

**Table 2:** Pigment content (pg·cell^−1^) in *Gambierdiscus* species isolated from Bahía de La Paz, Gulf of California.

Species	Strain Code	Chl *a*	Chl *c2*	Diadino	Dino	Diato	Peri	ββ-Car
*Gambierdiscus* cf. *caribeus*	GbSa-2	149.38	57.79	26.41	2.05	26.41	81.26	6.02
	GbSa-7	203.79	37.08	25.77	4.71	3.67	55.73	6.45
*Gambierdiscus* cf. *carpenteri*	GbSa-4	152.41	34.24	21.10	0.00	3.90	49.47	3.99
	GbSa-5	124.95	22.88	15.70	3.28	3.26	34.42	4.10
	GbSa-6	278.69	30.96	15.99	4.74	5.67	23.89	12.85

**Table 3:** Concentrations of amino acids (pg·cell^−1^) in *Gambierdiscus* species isolated from Bahía de La Paz, Gulf of California.

Species	Strain Code	Glu	Asp	Ser	His	Gly	Thr	Ala	Arg	Tyr	Val	Phe	Iso	Leu	Lys	Pro
*Gambierdiscus* cf. *caribeus*	Gbsa-2	17.41	15.85	13.52	Nd	8.31	14.95	12.84	17.70	18.71	15.20	17.36	16.53	16.24	15.22	17.78
	Gbsa-7	32.74	28.15	22.68	13.04	16.69	24.79	22.33	25.69	24.26	24.44	27.11	23.06	29.33	23.24	52.30
*Gambierdiscus* cf. *carpenteri*	Gbsa-4	36.39	30.93	26.31	Nd	18.66	28.91	27.53	30.63	29.47	28.48	30.68	27.27	32.20	26.58	18.10
	Gbsa-5	14.80	12.74	11.45	Nd	7.72	12.52	11.26	14.51	14.52	12.78	14.20	13.08	14.35	12.86	12.52
	Gbsa-6	30.94	27.14	21.12	Nd	15.04	23.83	22.13	25.23	24.93	23.26	24.86	23.13	25.26	21.99	24.14

Aspartic acid (Asp), glutamic acid (Glu), serine (Ser), histidine (His), glycine (Gly), threonine (Thr), alanine (Ala), arginine (Arg), tyrosine (Tyr), valine (Val), phenylalanine (Phe), Isoleucine (Iso) leucine (Leu), lysine (Lys), and Proline (Pro).

**Table 4:** Pigment composition detected in isolates of the genus *Gambierdiscus*.

Specie/Strain	Pigments	Method	Reference
*Gambierdiscus toxicus* Gambier Island	Chl *c*1, Chl *c*2, and Peri	TLC	[68]
*Gambierdiscus toxicus* Southern coast Florida	Chl *a*, Chl *c*1, Chl *c*2, Diadino, Dino, Peri, and ββ-car	TLC	[69]
*Gambierdiscus toxicus* Fourteen clones: Bermuda Great Issacs Light, Bahamas; Drifth Algae, Gulf Stream; Gingerbreads, Bahamas; Hawaii; Marathon key, Fl; Martnique, Caribbean; Virgin Gorda, V.I.	Chl *a*, Chl *c2*, Peri, Diadino, and Dino	TLC, HPLC	[70]
*Gambierdiscus toxicus* Caribean clone	Chl *a*	NMR	[71]
*Gambierdiscus toxicus* Three clones:	Chl *a*	HPLC-DAD	[72]
*Gambierdiscus excentricus* Canary Islands (NE Atlantic Ocean)	Chl *a*, Chl *a* allomer, Chl *c*1, Chl *c2*, Diadchr, Diadino, Dino, Diato, (MgDVP), Peri, Perid-ol, Peri-like, Pyrrho, and ββ-car	HPLC-DAD	[73]
*Gambierdiscus* sp.	Chl *a*, Chl *c*1, Chl *c*2, Diadino, Dino, and Peri	HPLC-DAD	[63]
*Gambierdiscus* cf. *caribaeus* *Gambierdiscus* cf. *carpenteri* Five clones: Bahía de La Paz, Gulf of California	Chl *a*, Chl *c*2, Diadino, Dino, Diato, Peri; and ββ-car Chl *a*, Chl *c*2, Diadino, Dino, Diato, Peri; and ββ-car	HPLC-DAD HPLC-DAD	This study This study

Chlorophyll *a* (Chl *a*), Chlorophyll *c*1 (Ch *c*1), Chlorophyll *c*2, Peridine (Peri), diadinoxantin (Diadino), dinoxantina (Dino), β, β-carotene (ββ-car), Chrophyll *a* allomer (Chl *a* allomer), diatoxanthin (Diato), diadinochrome (Diadchr), divinyl protochlorophyllide (MgDVP), peridinin-like Peridininol (Perid-ol), peridinin-like (Peri-like), and pyrrhoxanthin (Pyrrho).

**Table 5:** Amino acid composition detected in diverse species of dinoflagellates.

Dinoflagellate/Strain	Amino Acids	Method	Family	Reference
*Prorocentrum triestinum*	Asp, Ser, Thr, Glu, Pro, Gly, Ala, Cys, Val, Met, Iso, Leu, Tyr, Phe, Lys, His, Arg, Try		Prorocentraceae	[76]
*Prorocentrum minimum, Noctiluca scintillans*	His, Thr, Arg, Val, Phe, Iso; Leu, Lys, Asp, Glu, Ser, Gly, Ala, Tyr, Cys, Met, Try, and Pro	HSAA	Prorocentraceae, Noctilucaceae	[77]
*Aureodinium pigmentosum, Prorocentrum micans*, *Glenodinium foliaceum*, *Heterocapsa triquetra*, *Scrippsiella trochoidea*, *Alexandrium tamarense* y *Gymnodinium catenatum*	Glu, Gln, Arg, Tau, Gin/Glu	HPLC		[74]
*Heterocapsa rotundata* Shiwa Bay, Korea	His, Thr, Arg, Val, Phe, Iso; Leu, Lys, Asp, Glu, Ser, Gly, Ala, Tau, GABA, Tyr, and Pro	HPLC-FLD	Heterocapsaceae	[78]
*Ansanella granifera* Shiwa Bay, Korea	His, Thr, Arg, Val, Met, Phe, Iso; Leu, Lys, Asp, Glu, Ser, Gly, Ala, Tau, GABA, Tyr, and Pro		Suessiaceae	
*Alexandrium andersonii* Jinhae Bay, Korea	His, Thr, Arg, Val, Met, Phe, Iso; Leu, Lys, Asp, Glu, Ser, Gly, Ala, Tau, GABA, Tyr, and Pro		Ostreopsidaceae	
*Takayama tasmanica* Glenhaven, New Zeland	His, Thr, Arg, Val, Met, Phe, Iso; Leu, Lys, Asp, Glu, Ser, Gly, Ala, Tyr, and Pro		Brachidiniaceae	
*Tanayama helix* Tasman Sea, Australia	His, Thr, Arg, Val, Met, Phe, Iso; Leu, Lys, Asp, Glu, Ser, Gly, Ala, Tyr, and Pro		Brachidiniaceae	
*Gymnodnium smaydae* Shiwa Bay, Korea	His, Thr, Arg, Val, Met, Phe, Iso; Leu, Lys, Asp, Glu, Ser, Gly, Ala, Tau, GABA, Tyr, and Pro		Gymnodinaceae	
*Gambierdiscus* cf. *caribaeus* *Gambierdiscus* cf. *carpenteri* Five clones: Bahía de La Paz, Gulf of California	His, Thr, Arg, Val, Phe, Iso; Leu, Lys, Asp, Glu, Ser, Gly, Ala, Tyr, and Pro His, Thr, Arg, Val, Phe, Iso; Leu, Lys, Asp, Glu, Ser, Gly, Ala, Tyr, and Pro	HPLC-DAD HPLC-DAD	Ostreopsidaceae Ostreopsidaceae	This study This study

Cysthine (Cys), histidine (His), threonine (Thr), arginine (Arg), valine (Val), methionine (Met), phenylalanine (Phe), isoleucine (Iso), leucine (Leu), lysine (Lys), aspartic acid (Asp), glutamic acid (Glu), glutamine (Gln), serine (Ser), glycine (Gly), alanine (Ala), taurine (Tau), gamma-aminobutyric acid (GABA), tryptophan (Try), tyrosine (Tyr), and proline (Pro). HSAAA: High-speed amino acid analyzer; HPLC-FLD: high-performance liquid chromatography with fluorescence detection.

**Table 6:** Toxicity assays performed and principal marine toxins detected in *Gambierdiscus caribaeus* and *G. carpenteri* strains.

Specie/Strain	Origin	Assays Toxicity	Toxins Detected	Method	Reference
*Gambierdiscus caribaeus*					
Gam19, Jar 17 gam 20, Gam 4, SW gam 1, NCMA 1733, TC tow Gam 3, Norval Cay, SW gam 5, CBC gam1 NCMA 1651 Mexico Algae 1 gam 1 Jamaica Algae 1 gam 1 ETB Gam 6, Outfish 7-1, Outhfish 7-3 Jar 2 Tow 3, Keys jar 7 gam 7 ST1 C5 SJ3 07 BB gam 4 BRP gam 4, Coral cove gam 1 Dive 1 fa gam 1 WBHR 21 gam 2, WBHR 26 gam 1	Belize Gran Cayman) Cancun, Mexico Ocho ríos, Jamaica DryTortugas (Long key, Florida); St. Thomas St. Jhons Bathtub Beach, Florida Jupiter, Florida Ft. Pierce, Florida Flower Garden	HA	---	---	[119]
CBCGam1 CCMP1651 Dive1TA KeysJar7 MexicoAlgae1 SWGam5	Carrie Bow Cay Belize Gran Cayman Island, Caribbean Carrie Bow Cay, Belize Florida Keys, USA Cancún, Mexico Southwater Cay, Belize	CBA-N2a Average, within species toxicity (fg CTX3C eq cell^−1^ (0.66 ± 0.34, 51%))	---	---	[47]
BillHiGam8	Waikiki Beach, Honolulu, Hawaii	CBA-N2a and ELA (DSF: 1.6–1.0 fg CTX3C eq cell^−1)^ MSF: 5.3–1.0 pg MTX eq cell^−1^	---	---	[119]
CUB4A5	Cienfuegos, Cuba	RBA	---	---	[42]
10 strains Culture Collection of Harmful Microalgae of the Spanish Institute of Oceanography (CCVIEO)	Canary Islands	Neuro-2a Cell Assay, ELA (<LOD to 90.37 ± 15.89 fg CTX1B eq cell^−1^)	---	---	[120]
1 strain GCBG01	Micronesia Weizhou Island, Beibu Gulf of China	MBA Neuro-2a cells (0.54 fg CTX3C eq cell^−1^)	44-methylgambierone ---	LC-MS/MS ---	[121] [122]
BPAug08 and USVI-08	St. Thomas, US Virgin Islands	N2a–MTT Gambierone (0.6 ± 0.2 pg cell^−1^ and 9.8 ± 0.6 pg cell^−1^ 44-methylgambierone: 7.1 ± 4.2 pg cell^−1^)	Gambierone, 44-methylgambierone C-CTX-5	LC–HRMS	[24]
DiveIFM4 NOAA STIC5 NOAA	Atlantic Ocean	---	Gambierone, 44-methylgambierone	LC-MS/MS	[28]
CAWD301	Pohnpei	---	Gambieric acid A, methylgambierone, MTX-7 trace	LC-MS/MS	[18]
BPAug08 and USVI-08	St. Thomas, US Virgin Islands	---	C-CTX-5	NMR spectroscopy	[25]
*G.* cf. *caribaeus*/ GbSa-2 and GbSa-7	El Sauzoso, Gulf of California, Mexico	MBA, ARTOX, MFA (CTXs and MTXs-like activity) fRBA	---	---	This study
*Gambierdiscus carpenteri*					
Mixed PR gam 4NCMA 1654 GT4 PatH1jar 5 gam 3 WBHR21 ETB Exp 24 gam 1 Jamaica Algae 2 gam 1	Puerto Rico Guam Belize Oahu, Hawaii Flower Gardens Dry Tortugas Ocho Rios	Hemolytic assay	---	---	[119]
4 isolates	Cook islands, French Polynesia and Australia	MBA (LD_50_ 20– 38 mg kg^−1^)	44-methylgambierone in Cook island and French Polynesia strains; Australia island negative strains	LC-MS/MS	[123]
CAWD237 CAWD364	Australia	---	MTX-6 trace Gambierone, 44-methylgambierone, Gambieric acid A	LC-MS/MS	[18]
Bill Aruba Gam15 GT4 Jamaica Algae2Gam1 Mexico Algae2Gam1 WBHR21	Aruba, Caribbean Carrie Bow Cay Belize Ocho Rios, Jamaica, Caribbean Cancun, Mexico Flower Garden Banks Nat. Mar. Sanctuary (West Bank) Gulf of Mexico, USA	CBA-N2a Average, within species toxicity (fg CTX3C eq cell^−1^) (0.89 ± 0.41, 47%)	---	---	[48]
AWD237	New South Wales, Australia	MBA (LD_50_ 5.1–14.4 mg/Kg)	CTXs and MTXs no detected	LC-MS/MS	[123]
Tropical UTSH12C4 UTSH16C3 UTSH16A1 UTSH16D2	Heron Island Lagoon, Australia	Ca2+ Influx SH-SY5Y Cell FLIPR Bioassay MTX-like activity (3–4)	MTX-3	LC-MS/MS	[124]
Gam1BOL_080513	Bolinao, Pangasinan, Luzon Island Phillipnes	RBA (7.48 ± 0.49 pg PbTx eq cell^−1^)	---	---	[45]
NHA19 and NAH20	Anaho Bay (Nuku Hiva Island, Marquesas archipelago)	CBA-N2a Were nontoxic	---	---	[125]
Gam1BOL080513	Bolinao, Pangasinan	RBA	44-methylgambierone	UPLC-MS/MS	[27]
GCARBAPAZ3	Isla Gaviota, Gulf of California, Mexico	MBA (CTXs and MTXs-like activity) 5.9 mg kg^−1^ CTXs-like and 0.06 mg kg^−1^ MTX-like	---	---	[38]
*G.* c.f. *carpenteri*/ GbSa-4, GbSa-5, and GbSa-6	El Sauzoso, Gulf of California, Mexico	MBA, ARTOX, MFA (CTXs and MTXs-like activity) f -RBA	---	---	This study

HA: Hemolytic assay; CBA-N2a: neuroblastoma cytotoxicity assay; RBA: radioligand-receptor binding assay; ELA: erythrocyte lysis assay; MTT: 3-(4,5-dimethyl-2-thiazol)-2,5-diphenyl-2H-tetrazolium bromide (methylthiazolyldiphenyl-tetrazolium bromide) colorimetric assay; MBA: mouse bioassay; FLIPR bioassay: fluorescent imaging plate reader; ARTOX: artemia assay; MFA: marine fish assay; fRBA: fluorescent receptor binding assay; LC–HRMS; high-performance liquid chromatography/H nuclear magnetic resonance; LC-MS/MS: liquid chromatography coupled to tandem low-resolution mass spectrometry; NMR spectroscopy: nuclear magnetic resonance spectroscopy; UPLC-MS/MS: ultra-performance liquid chromatography/mass spectrometry.

## Data Availability

Data is contained within the article or Supplementary Materials.

## Supplementary Materials

The following supporting information can be downloaded at https://www.mdpi.com/article/10.3390/md22090422/s1, Table S1: Species of dinoflagellates of the genera Gambierdiscus and Fukuyoa in the Mexican Pacific and Gulf of California; Table S2. Gambierdiscus and Fukuyoa species recorded in the site collected; Figure S3. LM plate tabulation Gambierdiscus cf. caribaeus (GbSa-2); Figure S4. LM plate tabulation Gambierdiscus cf. carpenteri (GbSa-5); Figure S5. LM plate tabulation Gambierdiscus cf. carpenteri (GbSa-6); Figure S6. Growth curve of Gambierdiscus cf. caribaeus and Gambierdiscus cf. carpenteri; Figure S7. LM Gambierdiscus cf. carolineanus (GbEs-4); Figure S8. LM Gambierdiscus cf. toxicus (GbEs-3); Figure S9. LM Fukuyoa cf. yasumotoi (FyEs-1); Figure S10. Chromatogram of the amino acid proline; Figure S11. Sigmoid dose response curve as a percentage of total binding.

## References

[1] Chinain M., Gatti C.M.I., Darius H.T., Quod J.P., Tester P.A. (2021). Ciguatera poisonings: A global review of occurrences and trends. Harmful Algae.

[2] FAO, WHO (2020). Report of the Expert Meeting on Ciguatera Poisoning: Rome, 19–23 November 2018.

[3] Anderson D.M., Glibert P.M., Burkholder J.M. (2002). Harmful algal blooms and eutrophication: Nutrient sources, composition, and consequences. Estuaries.

[4] Glibert P.M., Anderson D.A., Gentien P., Granéli E., Sellner K.G. (2005). The global, complex phenomena of harmful algal blooms. Oceanography.

[5] Hallegraeff G.M. (2010). Ocean climate change, phytoplankton community responses, and harmful algal blooms: A formidable predictive challenge. J. Phycol..

[6] Gobler C.J., Doherty O.M., Hattenrath-Lehmann T.K., Griffith A.W., Kang Y., Litaker R.W. (2017). Ocean warming since 1982 has expanded the niche of toxic algal blooms in the North Atlantic and North Pacific oceans. Proc. Natl. Acad. Sci. USA.

[7] Heisler J., Glibert P.M., Burkholder J.M., Anderson D.M., Cochlan W., Dennison W.C., Dortch Q., Gobler C.J., Heil C.A., Humphries E. (2008). Eutrophication and harmful algal blooms: A scientific consensus. Harmful Algae.

[8] Griffith A.W., Gobler C.J. (2020). Harmful algal blooms: A climate change co-stressor in marine and freshwater ecosystems. Harmful Algae.

[9] Skinner M.P., Brewer T.D., Johnstone R., Fleming L.E., Lewis R.J. (2011). Ciguatera fish poisoning in the Pacific Islands (1998 to 2008). PLoS Negl. Trop. Dis..

[10] Gingold D.B., Strickland M.J., Hess J.J. (2014). Ciguatera fish poisoning and climate change: Analysis of National Poison Center data in the United States, 2001–2011. Environ. Health Perspect..

[11] Núñez-Vázquez E.J., Almazán-Becerril A., López-Cortés D.J., Heredia-Tapia A., Hernández-Sandoval F.E., Band-Schmidt C.J., Bustillos-Guzmán J.J., Gárate-Lizárraga I., García-Mendoza E., Salinas-Zavala C.A. (2019). Ciguatera in Mexico (1984–2013). Mar. Drugs.

[12] Parrilla-Cerrillo M.C., Vázquez-Castellanos J.L., Sáldate-Castañeda E.O., Nava-Fernández L.M. (1993). Brotes de toxiinfecciones alimentarias de origen microbiano y parasitario. Salud Pública Mex..

[13] Barton E.D., Tanner P., Turchen S.G., Tungen C.L., Manoguerra A., Clarck R.F. (1995). Ciguatera fish poisoning: A Southern California epidemic. West. J. Med..

[14] Lechuga-Devéze C., Sierra-Beltrán A. (1995). Documented case of ciguatera on the Mexican Pacific Coast. Nat. Toxins.

[15] Núñez-Vázquez E., Sierra-Beltrán A.P., Ochoa J.L. Presencia de Biotoxinas Tipo Ciguatoxinas en Peces Carnívoros de Baja California Sur. Proceedings of the XI Simposium Internacional de Biología Marina.

[16] Núñez-Vázquez E.J., Sierra-Beltrán A.P., and Ochoa J.L. Fish Poisoning in Mexico. Proceedings of the Abstracts of the VIII Conferencia Internacional sobre Algas Nocivas.

[17] Núñez-Vázquez E.J., Bustillos-Guzmán J., Heredia-Tapia A., Yasumoto T., Cruz-Villacorta A., Band-Schmidt C.J., Gárate-Lizárraga I., López-Cortés D., Hernández-Sandoval F.E., Ochoa J.L. (2009). Múltiples toxinas marinas en el hígado de *Mycteroperca prionura*, *M. rosacea* y *Lutjanus colorado* asociados a la ciguatera en la isla El Pardito, B.C.S. México. Resúmenes del III Taller sobre Florecimientos Algales Nocivos: Integración del Conocimiento Sobre Eventos de FAN en México.

[18] Murray J.S., Passfield E.M.F., Rhodes L.L., Puddick J., Finch S.C., Smith K.F., van Ginkel R., Mudge E.M., Nishimura T., Funaki H. (2024). Targeted Metabolite Fingerprints of Thirteen *Gambierdiscus*, Five *Coolia* and Two *Fukuyoa* Species. Mar. Drugs.

[19] Holmes M.J. (1998). *Gambierdiscus yasumotoi*, sp. nov. (Dinophyceae), a toxic benthic dinoflagellate from Southeastern Asia. J. Phycol..

[20] Gómez F., Qiu D., Lopes R.M., Lin S. (2015). *Fukuyoa paulensis* gen. et sp. nov., a New Genus for the Globular Species of the Dinoflagellate *Gambierdiscus* (Dinophyceae). PLoS ONE.

[21] Li Z., Park J.S., Kang N.S., Chomérat N., Mertens K.N., Gu H., Lee K.-W., Kim K.H., Baek S.H., Shin K. (2020). A new potentially toxic dinoflagellate *Fukuyoa koreansis* sp. nov. (*Gonyaulacales*, Dinophyceae) from Korean coastal waters: Morphology, phylogeny, and effects of temperature and salinity on growth. Harmful Algae.

[22] Wang D.Z., Xin Y.H., Wang M.H. (2022). *Gambierdiscus* and Its Associated Toxins: A Minireview. Toxins.

[23] Kohli G.S., Farrell H., Murray S.A., Botana L.M., Louzao C.M., Vilariño N. (2015). Gambierdiscus, the cause of ciguatera fish poisoning: An increased human health threat influenced by climate change. Climate Change and Marine and Freshwater Toxins.

[24] Mudge E.M., Miles C.O., Ivanova L., Uhlig S., James K.S., Erdner D.L., Fæste C.K., McCarron P., Robertson A. (2023). Algal ciguatoxin identified as source of ciguatera poisoning in the Caribbean. Chemosphere.

[25] Miles C.O., Burton I.W., Lewis N.I., Robertson A., Giddings S.D., McCarron P., Mudge E.M. (2024). Isolation of Caribbean Ciguatoxin-5 (C-CTX5) and confirmation of its structure by NMR spectroscopy. Tetrahedron.

[26] Flores-Holguín N., Salas-Leiva J.S., Núñez-Vázquez E.J., Tovar-Ramírez D., Glossman-Mitnik D. (2023). Exploring marine toxins: Comparative analysis of chemical reactivity properties and potential for drug discovery. Front. Chem..

[27] Malto Z.B.L., Benico G.A., Batucan J.D., De la Cruz J., Romero M.L.J., Azanza R.V., Salvador-Reyes L.A. (2022). Global mass spectrometric analysis reveals chemical diversity of secondary metabolites and 44-Methylgambierone production in Philippine *Gambierdiscus* strains. Front. Mar. Sci..

[28] Yon T., Réveillon D., Sibat M., Holland C.R., Litaker W., Nascimento S.M., Rossignoli A.E., Riobó P., Hess P., Bertrand S. (2024). Targeted and non-targeted mass spectrometry to explore the chemical diversity of the genus *Gambierdiscus* in the Atlantic Ocean. Phytochemistry.

[29] Sierra B.A., Cruz A., Núñez-Vázquez E.J., Del Villar L.M., Cerecero J., Ochoa J.L. (1998). An overview of the marine food poisoning in Mexico. Toxicon.

[30] Núñez-Vázquez E.J., Almazán-Becerril A., Heredia-Tapia A., Hernández-Becerril D.U., Troccoli-Ghinaglia L., Arredondo-Vega B.O., Herrera-Silveira J.A., Vázquez-Castellanos J.L., Ochoa J.L. Incidencia del envenenamiento por Ciguatera en Mexico. Proceedings of the 4th Reunión de Expertos en Envenenamientos por Animales Ponzoñosos.

[31] Okolodkov Y.B., Gárate-Lizárraga I. (2006). An annotated checklist of dinoflagellates (Dinophyceae) from the Mexican Pacific. Acta Botánica Mex..

[32] Gárate-Lizárraga I., Band-Schmidt C.J., Verdugo-Díaz G., Muñetón-Gómez M.J., Félix-Pico E., Funes-Rodríguez R., Gómez-Gutiérrez J., Palomares-García R. (2007). Dinoflagelados (Dinophyceae) del sistema lagunar Magdalena-Almejas. Estudios Ecológicos en Bahía Magdalena.

[33] Cortés-Altamirano R. (2012). Two new localities for *Gambierdiscus toxicus* in the Mexican Pacific. Harmful Algae News.

[34] Núñez-Vázquez E.J., Hernández-Sandoval F.E., Cordero-Tapia A., Almazán-Becerril A., Band-Schmidt C.J., Bustillos-Guzmán J., López-Cortés D., Salinas-Zavala C.A., Morales-Zárate M.V., Mejía-Rebollo A. Presencia de microalgas bénticas potencialmente nocivas y mortandad de peces asociadas al Parque Marino de Cabo Pulmo, B.C.S. Proceedings of the II Congreso Nacional de Sociedad Mexicana para el estudio de los Florecimientos Algales Nocivos.

[35] Garate-Lizárraga I., Okolodkov Y.B., Cortés-Altamirano R., García-Mendoza E., Quijano-Scheggia S.I., Olivos-Ortíz A., Núñez-Vázquez E.J. (2016). Microalgas formadoras de florecimientos algales en el Golfo de California. Florecimientos Algales Nocivos en Mexico.

[36] Okolodkov Y.B., Gárate-Lizárraga I., Martínez-Cruz M.E., Galicia-García C. Epibenthic dinoflagellates of the southern Gulf of California: Species composition and abundance (2015–2019). Proceedings of the 19th International Conference on Harmful Algae (ICHA 2021).

[37] Morquecho-Escamilla L., Reyes-Salinas A., Okolodkov Y.B., Gárate-Lizárraga I., Mazariegos-Villarreal A., Galicia-García C. Aislamiento y caracterización taxonómica de dinoflagelados epífitos en Bahía de La Paz e Isla San José, Golfo de California. Proceedings of the Memorias del IV Congreso Somefan y II Reunión Alen.

[38] Ramos-Santiago A.E., Band-Schmidt C.J., Leyva-Valencia I., Fernández-Herrera L.J., Núñez-Vázquez E.J., Okolodkov Y.B. (2024). *Gambierdiscus carpenteri* (Dinophyceae) from Bahía de La Paz, Gulf of California: Morphology, genetic affinities, and mouse toxicity. Bot. Mar..

[39] Litaker R.W., Vandersea M.W., Faust M.A., Kibler S.R., Chinain M., Holmes M.J., Holland W.C., Tester P.A. (2009). Taxonomy of *Gambierdiscus* including four new species, *Gambierdiscus caribaeus*, *Gambierdiscus carolinianus*, *Gambierdiscus carpenteri* and *Gambierdiscus ruetzleri* (Gonyaulacales, Dinophyceae). Phycologia.

[40] Díaz-Asencio L., Rojas Abrahantes G., Pérez Avilleira G., Chamero D., Moreira González A. (2023). Clave dicotómica para la identificación preliminar de las especies de *Gambierdiscus* y *Fukuyoa* reportadas en la región del Caribe. Rev. De Investig. Mar..

[41] Soler-Onís E., Fernández-Zabala J., Ojeda-Rodriguez A., Amorim A. (2016). Bloom of *Gambierdiscus caribaeus* in the temperate-subtropical waters of El Hierro, Canary Islands (north East Atlantic). Harmful Algae News.

[42] Díaz-Asencio L., Vandersea M., Chomérat N., Fraga S., Clausing R.J., Litaker R.W., Chamero-Lago D., Gómez-Batista M., Moreira-González A., Tester P. (2019). Morphology, toxicity and molecular characterization of *Gambierdiscus* spp. towards risk assessment of ciguatera in south central Cuba. Harmful Algae.

[43] Smith K.F., Rhodes L., Verma A., Curley B.G., Harwood D., Kohli G.S., Solomona D., Rongo T., Munday R., Murray S.A. (2016). A new *Gambierdiscus* species (Dinophyceae) from Rarotonga, Cook Islands: *Gambierdiscus cheloniae* sp. nov.. Harmful Algae.

[44] Kohli G.S., Murray S.A., Neilan B.A., Rhodes L.L., Harwood D.T., Smith K.F., Meyer L., Capper A., Brett S., Hallegraeff G.M. (2014). High abundance of the potentially maitotoxic dinoflagellate *Gambierdiscus carpenteri* in temperate waters of New South Wales, Australia. Harmful Algae.

[45] Vacarizas J., Benico G., Austero N., Azanza R. (2018). Taxonomy and toxin production of *Gambierdiscus carpenteri* (Dinophyceae) in a tropical marine ecosystem: The first record from the Philippines. Mar. Pollut. Bull..

[46] Chinain M., Gatti C.M., Roue M., Darius H.T., Subba Rao D.V. (2020). Ciguatera-causing dinoflagellates in the genera *Gambierdiscus* and *Fukuyoa*: Distribution, ecophysiology and toxicology. Dinoflagellates: Classification, Evolution, Physiology and Ecological Significance.

[47] Litaker R.W., Holland W.C., Hardison D.R., Pisapia F., Hess P., Kibler S.R., Tester P.A. (2017). Ciguatoxicity of Gambierdiscus and Fukuyoa species from the Caribbean and Gulf of Mexico. PLoS ONE.

[48] Tester P., Wickliffe L., Jossart J., Rhodes L., Enevoldsen H., Adachi M., Litaker W. (2018). Global distribution of the genera Gambierdiscus and Fukuyoa. Harmful Algae.

[49] Richlen M.L., Horn K., Uva V., Fachon E., Heidmann S.L., Smith T.B., Parsons M.L., Anderson D.M. (2024). Gambierdiscus species diversity and community structure in St. Thomas, USVI and the Florida Keys, USA. Harmful Algae.

[50] Xu Y., Richlen M.L., Liefer J.D., Robertson A., Kulis D., Smith T.B., Parsons M.L., Anderson D.M. (2016). Influence of environmental variables on *Gambierdiscus* spp. (Dinophyceae) growth and distribution. PLoS ONE.

[51] Loeffler C.R., Tartaglione L., Friedemann M., Spielmeyer A., Kappenstein O., Bodi D. (2021). Ciguatera Mini Review: 21st Century Environmental Challenges and the Interdisciplinary Research Efforts Rising to Meet Them. Int. J. Environ. Res. Public Health.

[52] Cementerio de Yates: Negligencia y Omisiones en la Bahía de La Paz Tras Paso de Norma. https://causanaturamedia.com/periodismo-cn/Cementerio-de-yates-negligencia-y-omisiones-en-la-bahia-de-La-Paz-tras-paso-de-Norma.

[53] Gobierno de BCS Sin Capacidad de Retirar Embarcaciones Varadas en La Paz: VCC. https://tribunademexico.com/gobierno-de-bcs-sin-capacidad/.

[54] Parsons M.L., Settlemier C.J., Ballauer J.M. (2011). An examination of the epiphytic nature of Gambierdiscus toxicus, a dinoflagellate involved in ciguatera fish poisoning. Harmful Algae.

[55] Parsons M.L., Brandt A.L., Ellsworth A., Leynse A.K., Rains L.K., Anderson D.M. (2017). Assessing the use of artificial substrates to monitor Gambierdiscus populations in the Florida Keys. Harmful Algae.

[56] Tester P.A., Kibler S.R., Holland W.C., Usup G., Vandersea M.W., Leaw C.P., Teen L.P., Larsen J., Mohammad-Noor N., Faust M.A. (2014). Sampling harmful benthic dinoflagellates: Comparison of artificial and natural substrate methods. Harmful Algae.

[57] Islas y Áreas Protegidas del Golfo de California. https://www.gob.mx/semarnat/articulos/islas-y-areas-protegidas-del-golfo-de-california-269050.

[58] Heredia-Tapia A., Arredondo-Vega B.O., NuñezVázquez E.J., Ochoa J.L. Partial biochemical characterization and toxicological evaluation of the dinoflagellate *Prorocentrum lima* (=*Exuviaella lima*) isolated from El Pardito Island in Baja California Sur, México. Proceedings of the Memorias 9th International Conference on Algal Blooms.

[59] Nascimento S.M., Purdie D.A., and Morris S. (2005). Morphology, toxin composition and pigment content of *Prorocentrum lima* strains isolated from a coastal lagoon in southern UK. Toxicon.

[60] David H., Laza-Martínez A., Kromkamp J.C., Orive E. (2018). Physiological response of *Prorocentrum lima* (Dinophyceae) to varying light intensities. FEMS Microbiol. Ecol..

[61] Wu H., Zhang H., Peng J., Zheng G., Lu S., Tan Z. (2023). Adaptive responses of geographically distinct strains of the benthic dinoflagellate, *Prorocentrum lima* (Dinophyceae), to varying light intensity and photoperiod. Harmful Algae.

[62] Zapata M., Freire J., Garrido J.L., Reguera B., Blanco J., Fernández M.L., Wyatt T. (1998). Pigment composition of several harmful algae as determined by HPLC using pyridine-containing mobile phase polymeric octadecylsilica column. Harmful Algae.

[63] Zapata M., Fraga S., Rodríguez F., Garrido J.L. (2012). Pigment-based chloroplast types in dinoflagellates. Mar. Ecol. Prog. Ser..

[64] Carreto J.I., Carignan M.O., Montoya N.G. (2001). Comparative studies on mycosporine-like amino acids, paralytic shellfish toxins and pigment profiles of the toxic dinoflagellates *Alexandrium tamarense*, *Acatenella* and *A. minutum*. Mar. Ecol. Prog. Ser..

[65] Carreto J.I., Seguel M., Montoya N.G., Clément A., Carignan M.O. (2001). Pigment profile of the ichthyotoxic dinoflagellate *Gymnodinium* sp. from a massive bloom in southern Chile. J. Plank. Res..

[66] Bustillos-Guzmán J., Garate-Lizarraga I., López-Cortés D., Hernández-Sandoval F.E. (2004). The use of pigment “fingerprints” in the study of harmful algal blooms. Rev. Biol. Trop..

[67] Liu S., Peng Y., Yu Z., Dong L.I., Deng C., Yu Z. (2014). HPLC pigment Profiles of 31 Harmful Algal Bloom Species Isolated from the Coastal Sea Areas of China. Ocean. Coast. Sea Res..

[68] Durand M., Berkaloff C. (1985). Pigments composition and chloroplast organization of *Gambierdiscus toxicus* Adachi and Fukuyo (Dinophyceae). Phycologia.

[69] Indelicato S.R., Watson D.A. (1986). Identification of the Photosynthetic Pigments of the Tropical Benthic Dinoflagellate *Gambierdiscus toxicus*. Mar. Fish. Rev..

[70] Bomber J.W., Tindall D.R., Venable C.W., Miller D.M., Graneli E. (1990). Pigment composition and low-ligth response of fourteen clones of *Gambierdiscus toxicus*. Toxic Marine Phytoplankton.

[71] Miller D.M., Tindall D.R., Venable C.W., Graneli E. (1993). NMR Spectroscopy of Clorophyll(s)-a isolated from *Gambierdiscus toxicus*. Toxic Marine Phytoplankton.

[72] Bustillos-Guzmán J., Diogene J., Reguera B., Blanco J., Fernández M.L., Wyatt T. (1998). Pigment content and toxicity in three strains of *Gambierdiscus toxicus*: Implications of cell volume differences. Harmful Algae.

[73] Fraga S., Rodríguez F., Caillaud A., Diogène J., Raho N., Zapata M. (2011). *Gambierdiscus excentricus* sp. nov. (Dinophyceae), a benthic toxic dinoflagellate from the Canary Islands (NE Atlantic Ocean). Harmful Algae.

[74] Flynn K., Flynn K.J., Jones K.J. (1993). Changes in dinoflagellate intracellular amino acids in response to diurnal changes in light and N supply. Mar. Ecol. Prog. Ser..

[75] Flynn K.J., Jones K.J., Raine R., Richard J., Flynn K. (1994). Use of intracellular amino acid analysis as an indicator of the physiological status of natural dinoflagellate populations. Mar. Ecol. Prog. Ser..

[76] Okaichi T. (1974). Significance of Amino Acid Composition of Phytoplankton and suspensoid in Marine Biological Production. Bull. Jap. Soc. Sc. Fish..

[77] Hayashi T., Suitani Y., Murakami M., Yamaguchi K., Konosu S., and Noda H. (1986). Protein and Amino Acid Compositions of Five Species of Marine Phytoplankton. Bull. Japan Soc. Sc. Fish..

[78] Lim A.S., Jeong H.J., Kim S.J., Ok J.E. (2018). Amino acids profiles of six dinoflagellate species belonging to diverse families: Possible use as animal feeds in aquaculture. Algae.

[79] Lewis R.L., Hallegraeff G.M., Anderson D.M., Cembella A.D., Enevoldsen H.O. (1995). Detection of ciguatoxins and related benthic dinoflagellate toxins: In vivo and in vitro methods. Manual on Harmful Marine Microalgae.

[80] FAO (2004). Marine Biotoxins.

[81] Bienfang P., Oben B., DeFelice S., Moeller P., Huncik K., Oben P., Bowen B. (2008). Ciguatera: The detection of neurotoxins in carnivorous reef fish from the coast of Cameroon, West Africa. Afr. J. Mar. Sci..

[82] Ley-Martínez T.C., Núñez-Vázquez E.J., Balart-Páez E.F., Poot-Delgado C.A., Band-Schmidt C.J., Almazán-Becerril A. Identificación y cuantificación de ciguatoxinas en peces carnívoros de la Península de Yucatán. Proceedings of the Memorias del IV Congreso Nacional de la SOMEFAN y II Congreso Latinoamericano de la ALEAN.

[83] Neves R.A.F., Fernandes T., Santos L.N.d., Nascimento S.M. (2017). Toxicity of benthic dinoflagellates on grazing, behavior and survival of the brine shrimp *Artemia salina*. PLoS ONE.

[84] Heredia-Tapia A., Arredondo-Vega B.O., Núñez-Vázquez E.J., Yasumoto T., Yasuda M., Ochoa J.L. (2002). Isolation of *Prorocentrum lima* (Syn. *Exuviaella lima*) and diarrhetic shellfish poisoning (DSP) risk assessment in the Gulf of California, Mexico. Toxicon.

[85] Ajuzie C.C. (2007). Palatability and fatality of the dinoflagellate *Prorocentrum lima* to *Artemia salina*. J. Appl. Phycol..

[86] Faimali M., Giussani V., Piazza V., Garaventa F., Corrà C., Asnaghi V., Privitera D., Gallus L., Cattaneo-Vietti R., Mangialajo L. (2012). Toxic effects of harmful benthic dinoflagellate *Ostreopsis ovata* on invertebrate and vertebrate marine organisms. Mar. Environ. Res..

[87] Gong Y., Zhang K., Geng N., Wu M., Yi X., Liu R., Challis J.K., Codling G., Xu E.G., Giesy J.P. (2021). Molecular mechanisms of zooplanktonic toxicity in the okadaic acid-producing dinoflagellate *Prorocentrum lima*. Environ. Pollut..

[88] Yan M., Mak M.Y.L., Cheng J., Li J., Gu J.R., Leung P.T.Y., Lam P.K.S. (2020). Effects of dietary exposure to ciguatoxin P-CTX-1 on the reproductive performance in marine medaka (*Oryzias melastigma*). Mar. Pollut. Bull..

[89] Li L., Chen S., Xu S.Y., Li D.W., Li H.Y., Yang W.D. (2022). Toxicity and underlying mechanism of the toxic dinoflagellate *Gambierdiscus caribaeus* to the fish *Oryzias melastigma*. Ecotoxicol. Environ. Saf..

[90] Helfrich P., Bannerg A. (1963). Experimental Induction of Ciguatera Toxicity in Fish through Diet. Nature.

[91] Davin W.T., Kohler C.C., Tindall D.R. (1986). Effects of ciguatoxins on the bluehead. Trans. Am. Fish. Soc..

[92] Davin W.T., Kohler C.C. (1988). Ciguatera toxins adversely affect piscivorous fishes. Trans. Am. Fish. Soc..

[93] Lewis R.J. (1992). Ciguatoxins are potent ichthyotoxins. Toxicon.

[94] Magnelia S.J., Kohler C.C. (1992). Acanthurids Do Not Avoid Consuming Cultured Toxic Dinoflagellates yet Do Not Become Ciguatoxic. Trans. Am. Fish. Soc..

[95] Quod J. (1983). Action de la Ciguatoxine sur Léquilibrie de Distribution Hydroionique et sur la Perméabilité au Na^+^ du Muscle Blanc Dún Téléostéen, Chelon Labrosus.

[96] Capra M.F., Cameron J., Flowers A.E., Coombe I.F., Blanton C.G., Hahn S.T. The Effects of Ciguatoxin on Teleosts. Proceedings of the 6th International Coral Reef Congress.

[97] Durand-Clement M., Amade P., Puiseux-Dao S., Stadier T., Karamanos Y., Verdus M.C., Mollion J., Christiaen D. (1988). Induction of toxicity in fishes fed with cultures of the ciguateric dinoflagellate *Gambierdiscus toxicus*. Proceedings of the 4th International Meeting of the SAA.

[98] Kohler C.C., Paleudis G.A., Tindall D.R. (1989). Behavior abnormalities displayed by ocean surgeon following consumption of ciguatoxigenic dinoflagellates. Proc. Assn. Island Mar. Lab. Carib..

[99] Kelly A.M., Kohler C.C., Tindall D.R. (1992). Are crustaceans linked to the ciguatera food chain?. Environ. Biol. Fish..

[100] Kohler C.C., Kholer S., Gerace D.T. (1998). Ciguatera tropical fish poisoning: What’s happening in the food chain. Proceedings of the 26th Meeting of the Association of Marine Laboratories of the Caribbean.

[101] González G., Brusle J., Crespo S. (1994). Ultrastructural alterations of cabrilla sea bass *Serranus cabrilla* liver related to experimental *Gambierdiscus toxicus* (dinoflagellate) ingestion. Dis. Aqua. Org..

[102] Edmunds J.S., McCarthy R.A., Ramsdell J.S. (1999). Ciguatoxin reduces larval survivability in finfish. Toxicon.

[103] Mak L.Y., Li J., Liu C.N., Cheng S.H., Lam P.K.S., Cheng J., Chan L.L. (2017). Physiological and behavioural impacts of Pacific ciguatoxin-1 (P-CTX-1) on marine medaka (*Oryzias melastigma*). J. Hazard. Mater..

[104] Clausing R.J., Ben Gharbia H., Sdiri K., Sibat M., Rañada-Mestizo M.L., Lavenu L., Hess P., Chinain M., Bottein M.-Y.D. (2024). Tissue Distribution and Metabolization of Ciguatoxins in an Herbivorous Fish following Experimental Dietary Exposure to *Gambierdiscus polynesiensis*. Mar. Drugs.

[105] Yan M., Leung P.T., Ip J.C., Cheng J.P., Wu J.J., Gu J.R., Lam P.K. (2017). Developmental toxicity and molecular responses of marine medaka (*Oryzias melastigma*) embryos to ciguatoxin P-CTX-1 exposure. Aquat. Toxicol..

[106] Leite I.D.P., Sdiri K., Taylor A., Viallon J., Gharbia H.B., Mafra Júnior L.L., Swarzenski P., Oberhaensli F., Darius H.T., Chinain M. (2021). Experimental Evidence of Ciguatoxin Accumulation and Depuration in Carnivorous Lionfish. Toxins.

[107] Galarza J.R., Edwards R.A., Baden D.G., Tosteson T.R. (1993). Effect of Barracuda Ciguatoxins on Pigment Granule Aggregation in Teleost Melanophores. Bull. De La Soc. De Pathol. Exot..

[108] Igarashi T., Aritake S., Yasumoto T. (1999). Mechanisms underlying the hemolytic and ichthyotoxic activities of maitotoxin. Nat. Toxins.

[109] Murata M., Naoki H., Iwashita T., Matsunaga S., Sasaki M., Yokoyama A., Takeshi Y. (1993). Structure of Maitotoxin. J. Am. Chem. Soc..

[110] Nicolaou K.C., Frederick M.O., Aversa R.J. (2008). The continuing saga of the marine polyether biotoxins. Angew. Chem. Int. Ed. Engl..

[111] Kohli G.S., Papiol G.G., Rhodes L.L., Harwood D.T., Selwood A., Jerrett A., Murray S.A., Neilan B.A. (2014). A feeding study to probe the uptake of Maitotoxin by snapper (*Pagrus auratus*). Harmful Algae.

[112] Yasumoto T., Bagnis R., Vernoux J.P. (1976). Toxicity of the surgeonfishes. II. Properties of the principal water-soluble toxin. Bull. Jpn. Soc. Fish..

[113] Takahishi M., Tatsumi M., Ohizumi Y., Yasumoto T. (1983). Ca^2+^ channel activation function of maitotoxin the most potent marine toxin known, in clonal rat pheochromocytoma cells. J. Biol. Chem..

[114] Murata M., Yasumoto T. (2000). The structure elucidation and biological activities of high molecular weight algal toxins: Maitotoxin, prymnesins and zooxanthellatoxins. Nat. Prod. Rep..

[115] Terao K., Ito E., Igarashi T., Aritake S., Seki T., Satake M., Yasumoto T., Yasumoto T., Oshima Y., Fukuyo Y. (1996). Effects of Prymnesin, Maitotoxin and Gymnomidine on the structure of Gills of small fish Akahire, *Tanichtys albonubes*. Lin. Harmful and Toxic Algal Blooms.

[116] Landsberg J.H. (1995). Tropical reef fish disease outbreaks and mass mortalities in Florida: What is the role of dietary biological toxins?. Dis. Aquat. Org..

[117] McCall J.R., Jacocks H.M., Niven S.C., Poli M.A., Baden D.G., Bourdelais A.J. (2014). Development and utilization of a fluorescence-based receptor-binding assay for the site 5 voltage-sensitive sodium channel ligands brevetoxin and ciguatoxin. J. AOAC Int..

[118] Pisapia F., Holland W.C., Hardison D.R., Litaker R.W., Fraga S., Nishimura T., Adachi M., Nguyen-Ngoc L., Séchet V., Amzil Z. (2017). Toxicity screening of 13 *Gambierdiscus* strains using neuro-2a and erythrocyte lysis bioassays. Harmful Algae.

[119] Holland W.C., Litaker R.W., Tomas C.R., Kibler S.R., Place A.R., Davenport E.D., Tester P.A. (2013). Differences in the toxicity of six *Gambierdiscus* (Dinophyceae) species measured using an in vitro human erythrocyte lysis assay. Toxicon.

[120] Rossignoli A.E., Tudó A., Bravo I., Díaz P.A., Diogène J., Riobó P. (2020). Toxicity Characterisation of *Gambierdiscus* Species from the Canary Islands. Toxins.

[121] Murray J.S., Nishimura T., Finch S.C., Rhodes L.L., Puddick J., Harwood D.T., Larsson M.E., Doblin M.A., Leung P., Yan M. (2020). The role of 44-methylgambierone in ciguatera fish poisoning: Acute toxicity, production by marine microalgae and its potential as a biomarker for *Gambierdiscus* spp.. Harmful Algae.

[122] Xu Y., He X., Lee W.H., Chan L.L., Lu D., Wang P., Tao X., Li H., Yu K. (2021). Ciguatoxin-Producing Dinoflagellate *Gambierdiscus* in the Beibu Gulf: First Report of Toxic *Gambierdiscus* in Chinese Waters. Toxins.

[123] Munday R., Murray S., Rhodes L., Larsson M., Harwood D. (2017). Ciguatoxins and Maitotoxins in extracts of sixteen *Gambierdiscus* isolates and one *Fukuyoa* isolate from the South Pacific and their toxicity to mice by intraperitoneal and oral administration. Mar. Drugs.

[124] Larsson M., Laczka O., Harwood D., Lewis R., Himaya S., Murray S., Doblin M. (2018). Toxicology of *Gambierdiscus* spp. (Dinophyceae) from tropical and temperate Australian waters. Mar. Drugs.

[125] Darius H.T., Roué M., Sibat M., Viallon J., Gatti C.M., Vandersea M.W., Tester P.A., Litaker R.W., Amzil Z., Hess P. (2018). Toxicological Investigations on the Sea Urchin *Tripneustes gratilla* (Toxopneustidae, Echinoid) from Anaho Bay (Nuku Hiva, French Polynesia): Evidence for the Presence of Pacific Ciguatoxins. Mar. Drugs.

[126] Darius H.T., Revel T., Viallon J., Sibat M., Cruchet P., Longo S., Hardison D.R., Holland W.C., Tester P.A., Litaker R.W. (2022). Comparative Study on the Performance of Three Detection Methods for the Quantification of Pacific Ciguatoxins in French Polynesian Strains of *Gambierdiscus polynesiensis*. Mar. Drugs.

[127] Estevez P., Oses-Prieto J., Castro D., Penin A., Burlingame A., Gago-Martinez A. (2024). First Detection of Algal Caribbean Ciguatoxin in Amberjack Causing Ciguatera Poisoning in the Canary Islands (Spain). Toxins.

[128] Lluch-Cota S., Aragón-Noriega E.A., Arreguín-Sánchez F., Aurioles-Gamboa D., Bautista-Romero J.J., Brusca R.C., Cervantes-Duarte R., Cortés-Altamirano R., Del-Monte-Luna P., Esquivel-Herrera A. (2007). The Gulf of California: Review of ecosystem status and sustainability challenges. Prog. Oceanogr..

[129] Brusca R.C., Findley L.T., Hastings P.A., Hendrickx M.E., Torre J., van der Heiden A.M., Cartron J.L.E., Ceballos G., Felger R.S. (2005). Macrofaunal diversity in the Gulf of California. Biodiversity, Ecosystems, and Conservation in Northern Mexico.

[130] Brusca R.C. (2012). The Gulf of California: Biodiversity and Conservation. Arizona-Sonora Desert Museum Studies in Natural History.

[131] Gómez-Cabrera I. (2012). Pesca Deportiva y Pesca Ribereña en Baja California Sur, México: Comparación del Valor Económico. Ph.D. Thesis.

[132] Gómez-Cabrera I.D., Ivanova-Boncheva A. (2013). Valor económico de la pesca deportiva como fuente principal de atracción turística en Los Cabos, Baja California Sur, México. TURYDES Tur. Y Desarro. Local Sosten..

[133] Keller M.D., Selvin R.C., Claus W., Guillard R.R.L. (1987). Media for the culture of oceanic ultraphytoplankton. J. Phycol..

[134] Guillard R.R.L., Ryther J.H. (1962). Studies of marine planktonic diatoms. I. Cyclotella nana Hustedt, and Detonula confervacea (Cleve) Gran. Can. J. Microbiol..

[135] Guillard R.R. (1975). Culture of phytoplankton for feeding marine invertebrates. Culture of Marine Invertebrate Animals.

[136] Fritz L., Triemer R.E. (1985). A rapid simple technique utilizing Calcofluor White M28 for the visualization of dinoflagellate thecal plates. J. Phycol..

[137] Vidussi F., Claustre H., Bustillos-Guzmán J., Cailleau C., Marty J.C. (1996). Rapid HPLC method for determination of phytoplankton chemotaxinomic pigments: Separation of chlorophyll a from divinyl-chlorophyll a and zeaxanthin from lutein. J. Plankton Res..

[138] Montoura R.F.C., Repeta D., Jeffrey S.W., Mantoura R.F.C., Wright S.W. (1997). Calibration methods for HPLC. Phytoplankton Pigments in Oceanography: Guidelines to Modern Methods.

[139] Gratzfeld-Huesgen A. (1999). Sensitive and Reliable Amino Acid Analysis in Protein Hydrolysates Using the Agilent 1100 Series HPLC. Agilent Technologies. Publication Number 5968–5658E. https://channel.gimitec.com/sites/default/files/59685658.pdf.

[140] AOAC (2000). AOAC Official Method 994.12 Amino Acids in Feeds.

[141] Chinain M., Darius H.T., Ung A., Cruchet P., Wang Z., Ponton D., Laurent D., Pauillac S. (2010). Growth and toxin production in the ciguatera-causing dinoflagellate *Gambierdiscus polynesiensis* (Dinophyceae) in culture. Toxicon.

[142] Satake M., Murata M., Yasumoto T. (1993). The structure of CTX3C, a ciguatoxin congener isolated from cultured *Gambierdiscus toxicus*. Tetrahedr. Lett..

[143] Holmes M.J., Lewis R.J. (1994). Purification and characterisation of large and small maitotoxins from cultured *Gambierdiscus toxicus*. Nat. Toxins.

