# Supplementary material for: Morphological, Toxicological, and Biochemical Characterization of Two Species of Gambierdiscus from Bahía de La Paz, Gulf of California
Source: Mar Drugs. 2024 Sep 16;22(9):422. doi: 10.3390/md22090422 (PMC11433345; doi:10.3390/md22090422)
Supplement: Supplementary file 1 [file marinedrugs-22-00422-s001.zip › marinedrugs-3159571-supplementary.pdf]

marine drugs

Supplementary Materials

Table S1. Species of dinoflagellates of the genera *Gambierdiscus* and *Fukuyoa* in the Mexican Pacific and Gulf of California

Species	Site/Date	Reference
Gambierdiscus toxicus	Isla El Pardito, GC/1997	[29,30]
	Bahía Magdalena, PO and Bahía de La Paz, GC/2005	[31]
	Bahía Magdalena, PO/	[32]
	Isla Isabel and Isla Socorro, PO/2011	[33]
Gambierdiscus spp	Cabo Pulmo, GC/2010-2011	[34]
	Southern of GC/2016	[35]
	Bahía de La Paz, GC/2015, 2016, 2018, 2019	[36]
Fukuyoa sp.	Bahía de La Paz, GC/2015, 2016, 2018, 2019	[36]
Gambierdiscus cf. *caroli-nianus*	Isla San José and Bahía de La Paz, GC/2011, 2014, 2016	[37]
Gambierdiscus cf. *carpenteri*	Isla San José and Bahía de La Paz, GC/2011, 2014, 2016, Isla Gaviota	[37,38]
Fukuyoa yasumotoi	Isla San José and Bahía de La Paz, GC/2011, 2014, 2016	[37]

GC: Gulf of California; PO: Pacific Ocean

Table S2. *Gambierdiscus* and *Fukuyoa* species recorded in the site collected

**Specie**	**Location**	**Substrate**
Gambierdiscus cf. *caribaeus*	El Sauzoso	Coral reef
Gambierdiscus cf. *carpenteri*		Macroalgae *Padina* and *Dyctiota*
Gambierdiscus cf. *carpenteri*	El Conchalito Beach	Sandy sediment
Gambierdiscus cf. *carpenteri*	El Coromuel Beach	Coral reef
Gambierdiscus cf. *carpenteri*	El Caimancito Beach	Artificial substrate (old Marine Bridge piling)
Gambierdiscus cf. *carolineanus*	Espiritu Santo Island	Red and brown macroalgae adding to artificial substrate (Sunken Ship)
Gambierdiscus cf. *carpenteri*	Espiritu Santo Island	Red and brown macroalgae
Gambierdiscus cf. *toxicus*	Espiritu Santo Island	Rooky reef and artificial substrate (Sunken Ship)
Fukuyoa cf. *yasumotoi*	Espiritu Santo Island	Red and brown macroalgae adding to artificial substrate (Sunken Ship)

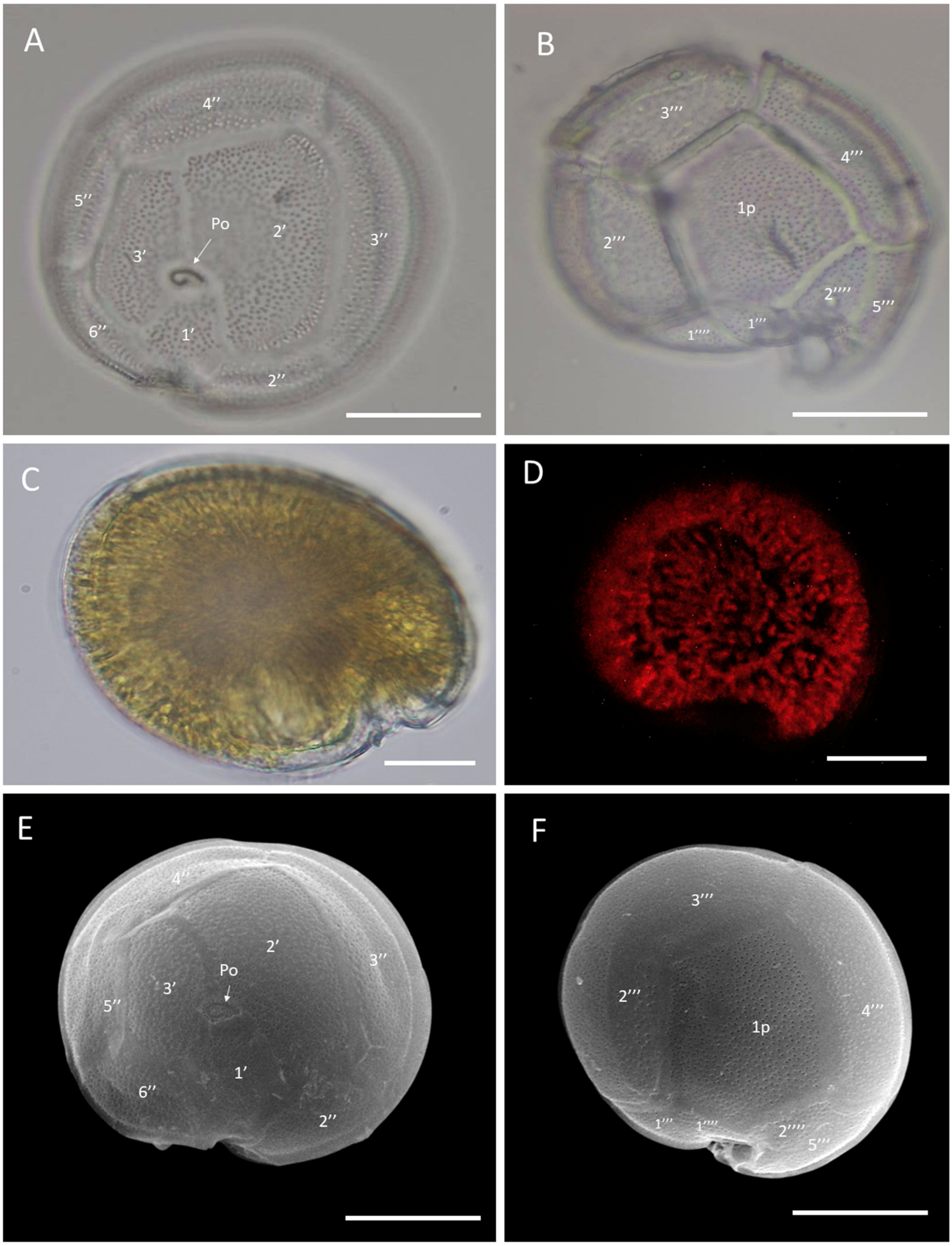

Figure S3. LM plate tabulation of *Gambierdiscus* cf. *caribaeus* (GbSa-2). (A) Apical view, (B) Antapical view, (C) Live cell, (D) Epifluorescence of chloroplast, (E) SEM plate tabulation Apical view, (F) SEM Antapical view. Scale bars: (A, B, and E) 50 µm, (C, D) 30 µm, and (F, E) 50 µm.

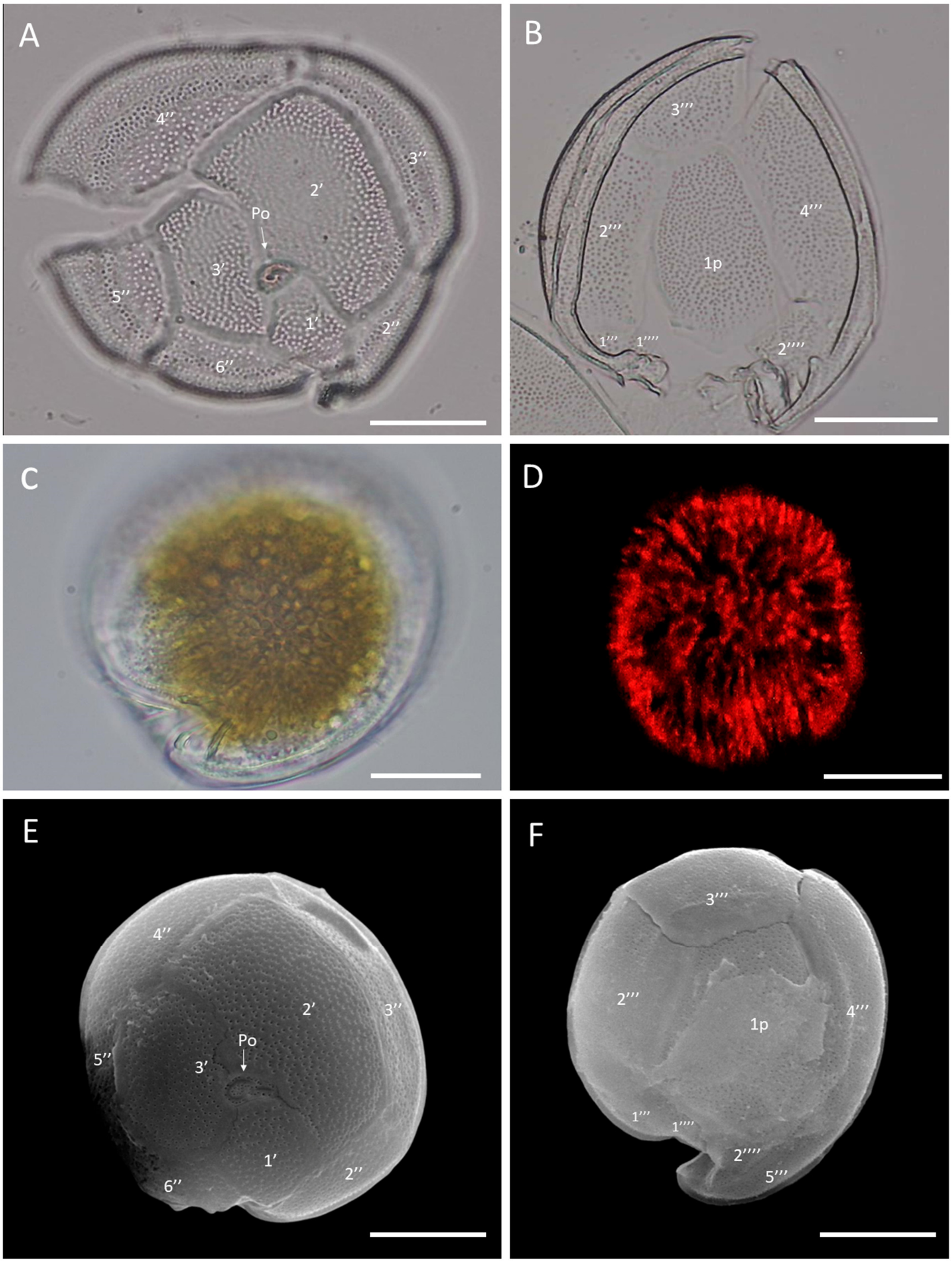

Figure S4. LM plate tabulation *Gambierdiscus* cf. *carpenteri* (GbSa-5). (A) Apical view, (B) Antapical view, (C) Live cell, (D) Epifluorescence of chloroplast, (E) SEM plate tabulation Apical view, (F) SEM Antapical view. Scale bars: (A, B, and E) 50 µm, (C, D) 30 µm, and (F, E) 50 µm.

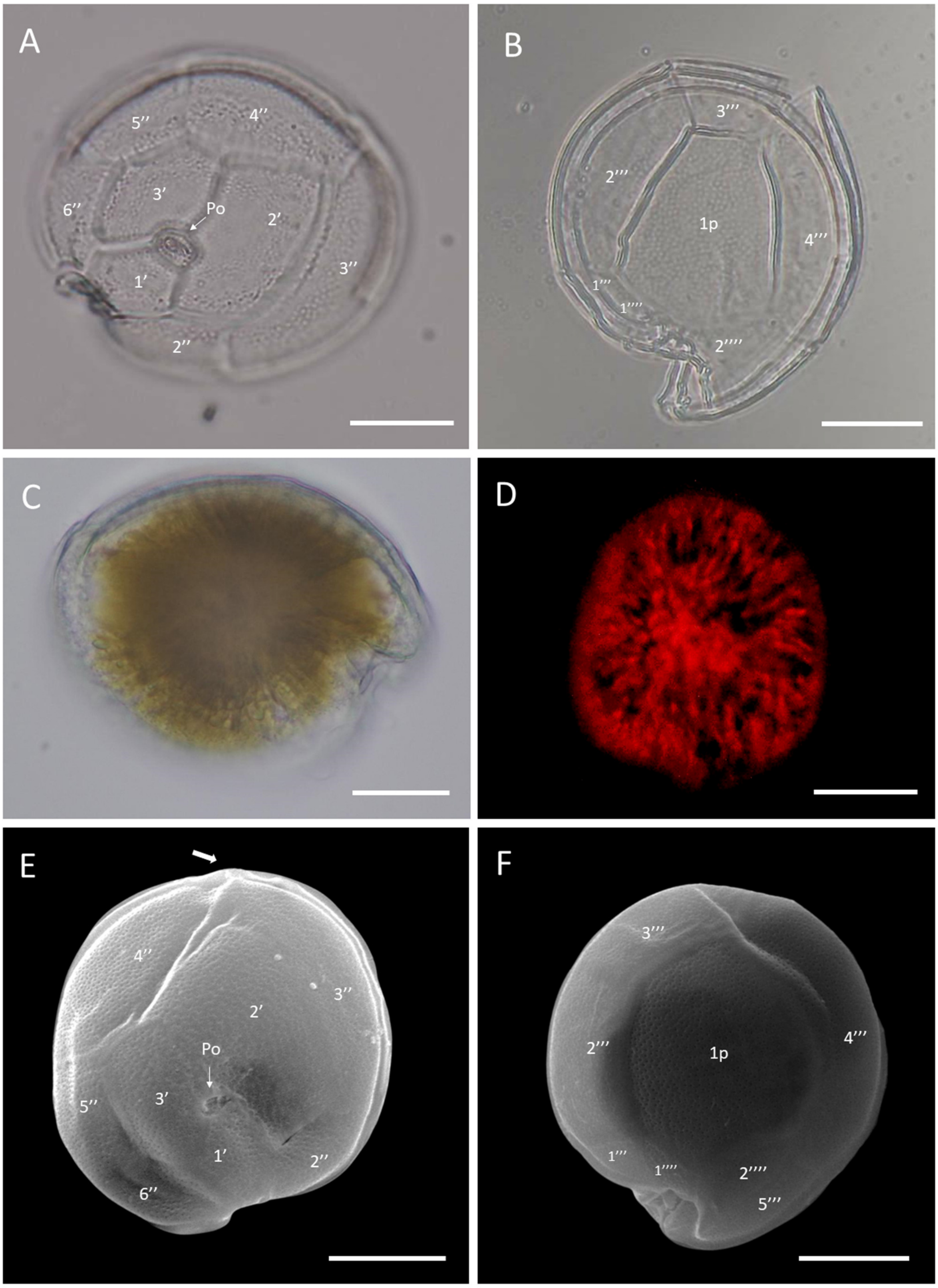

Figure S5. LM plate tabulation *Gambierdiscus* cf. *carpenteri* (GbSa-6). (A) Apical view, (B) Antapical view, (C) Live cell, (D) Epifluorescence of chloroplast, (E) SEM plate tabulation apical view, (F) Antapical view. Scale bars: (A, B, and E) 50 µm, (C, D) 30 µm, and (F, E) 50 µm.

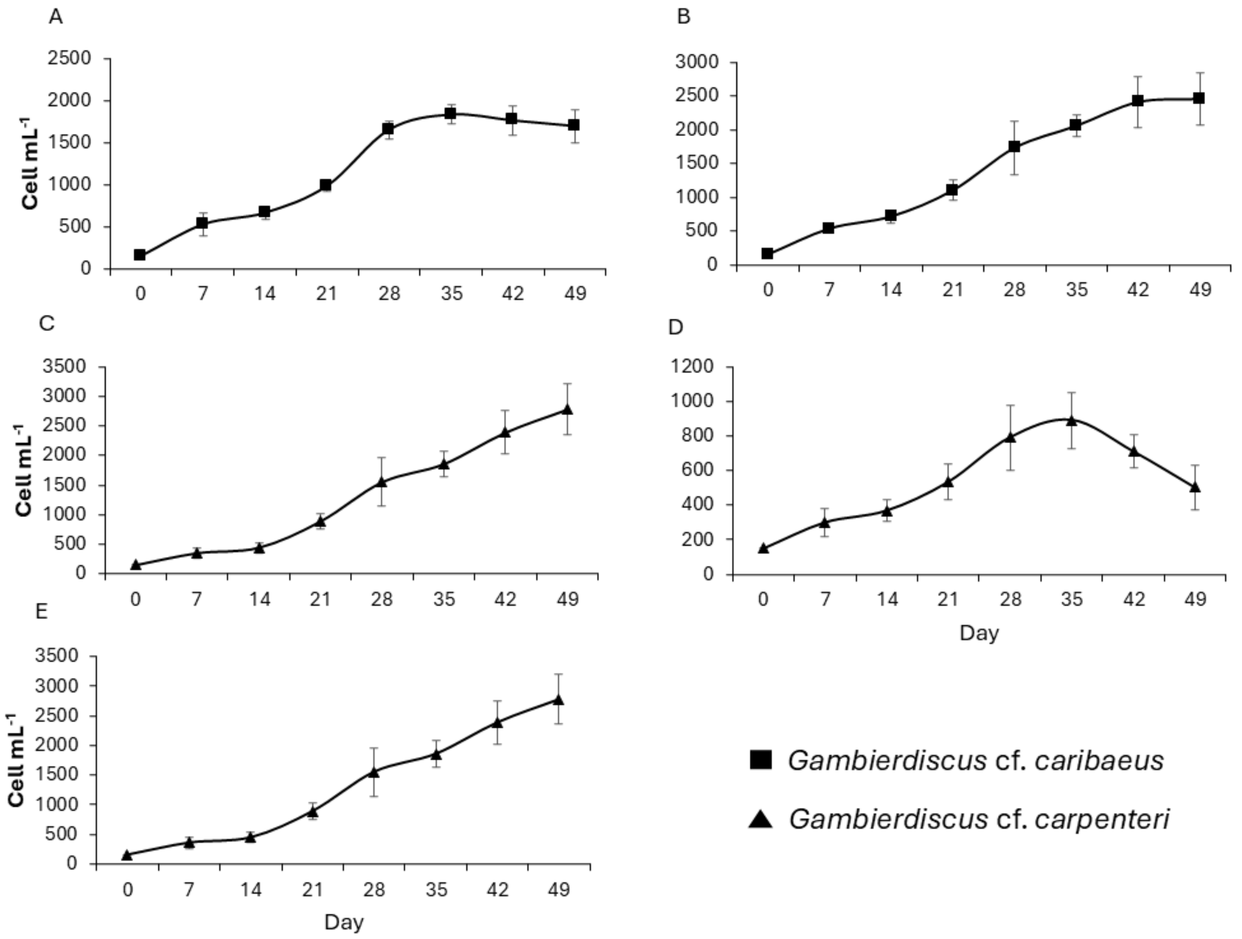

Figure S6. Growth curve of *Gambierdiscus* cf. *caribaeus* (A) GbSa-2 and (B) GbSa-7 and *Gambierdiscus* cf. *carpenteri* (C) GbSa-4, (D) GbSa-5, and (E) GbSa-6.

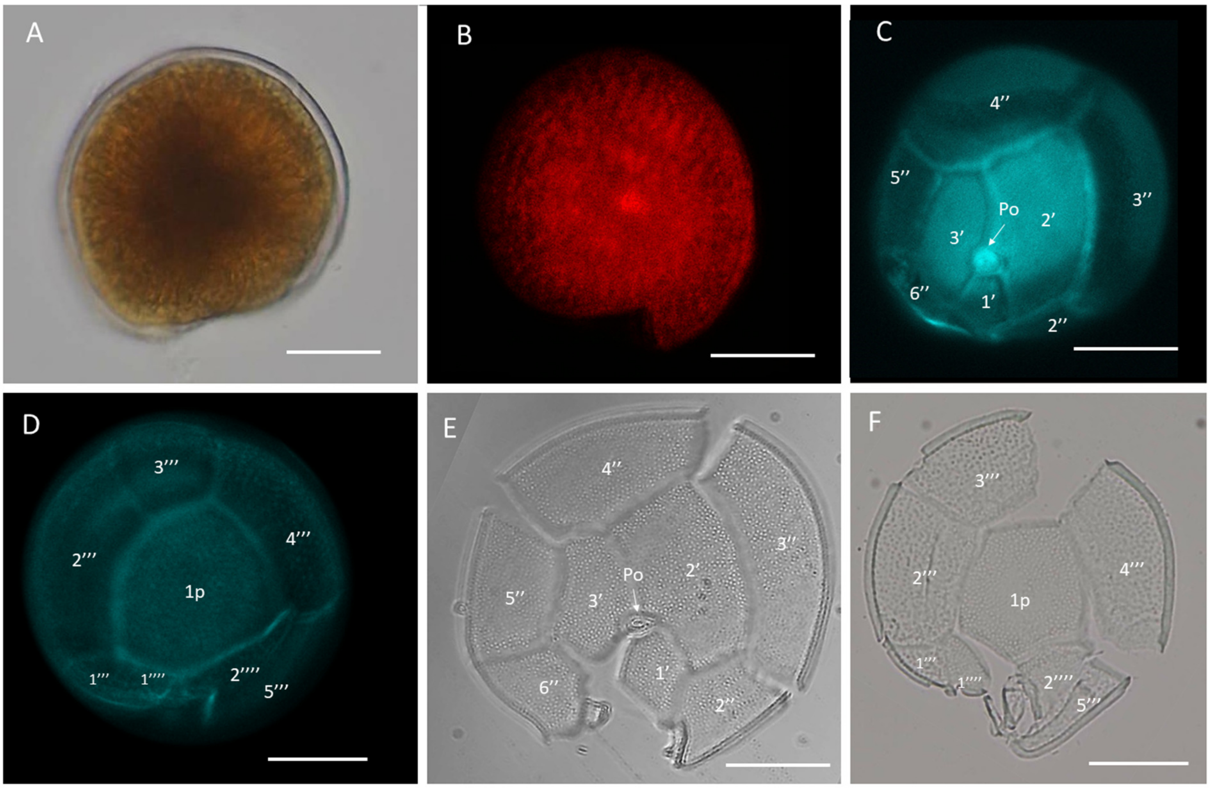

Figure S7. LM *Gambierdiscus* cf. *carolineanus* (GbEs-4). (A) Live cell, (B) Epifluorescence of chloroplast, (C) Plate tabulation apical view stained with fluorescent brightener 28, (E) apical view, (D) Plate tabulation apical view, (F) Antapical view. Scale bars: 40 µm.

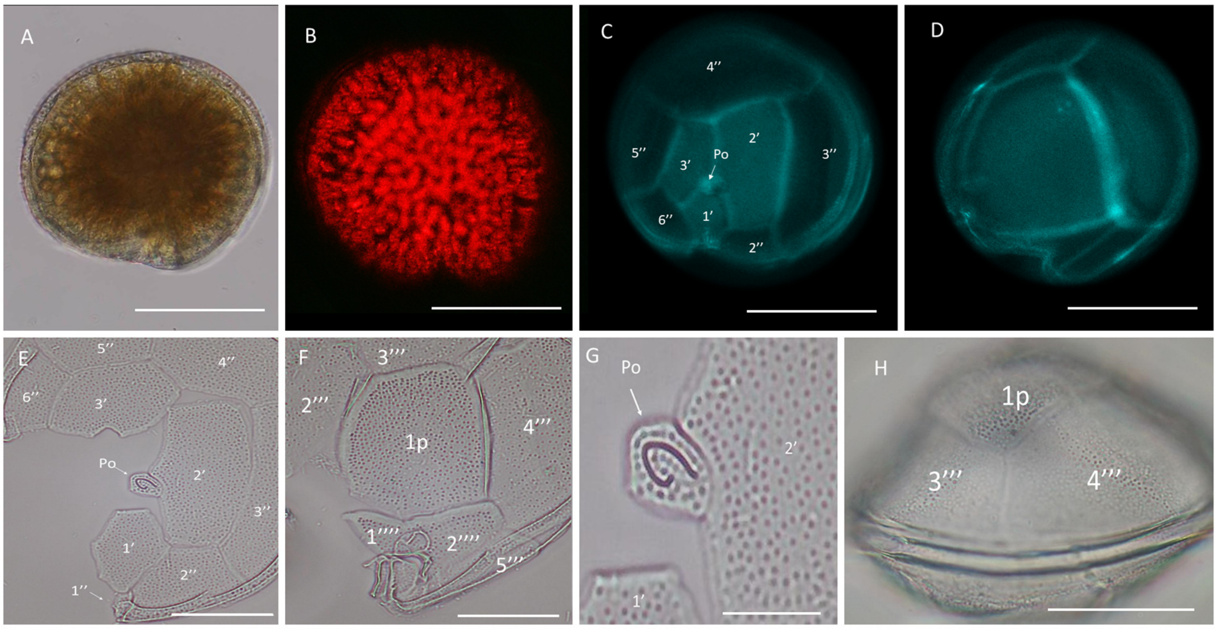

Figure S8. LM *Gambierdiscus* cf. *toxicus* (GbEs-3). (A) Live cell, (B) Epifluorescence of chloroplast, (C) Plate tabulation apical view stained with fluorescent brightener 28, (E) apical view, (D) Plate tabulation apical view, (F) Antapical view, (G) Po plante, (H) Dorsal view. Scale bars: (A-F and H) 50 µm and (G) 20 µm.

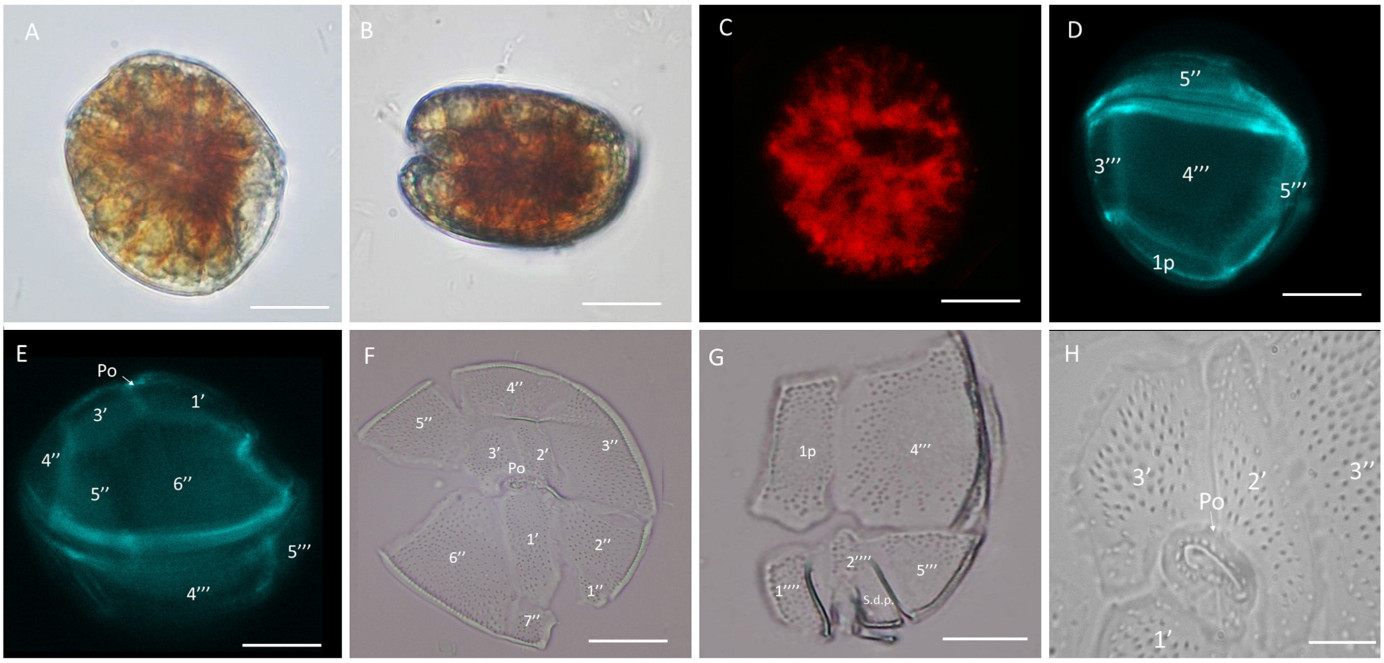

Figure S9. LM *Fukuyoa* cf. *yasumotoi* (FyEs-1). Live cell (A) lateral view, (B) antapical view, (C) Epifluorescence of chloroplast, (D) Plate tabulation lateral view stained with fluorescent brightener 28, (E) Dorso-lateral view, (F) Plate tabulation apical view, (G) Antapical view, (H) Po plate. Scale bars: (A-G) 10 µm and (G) 20 µm.

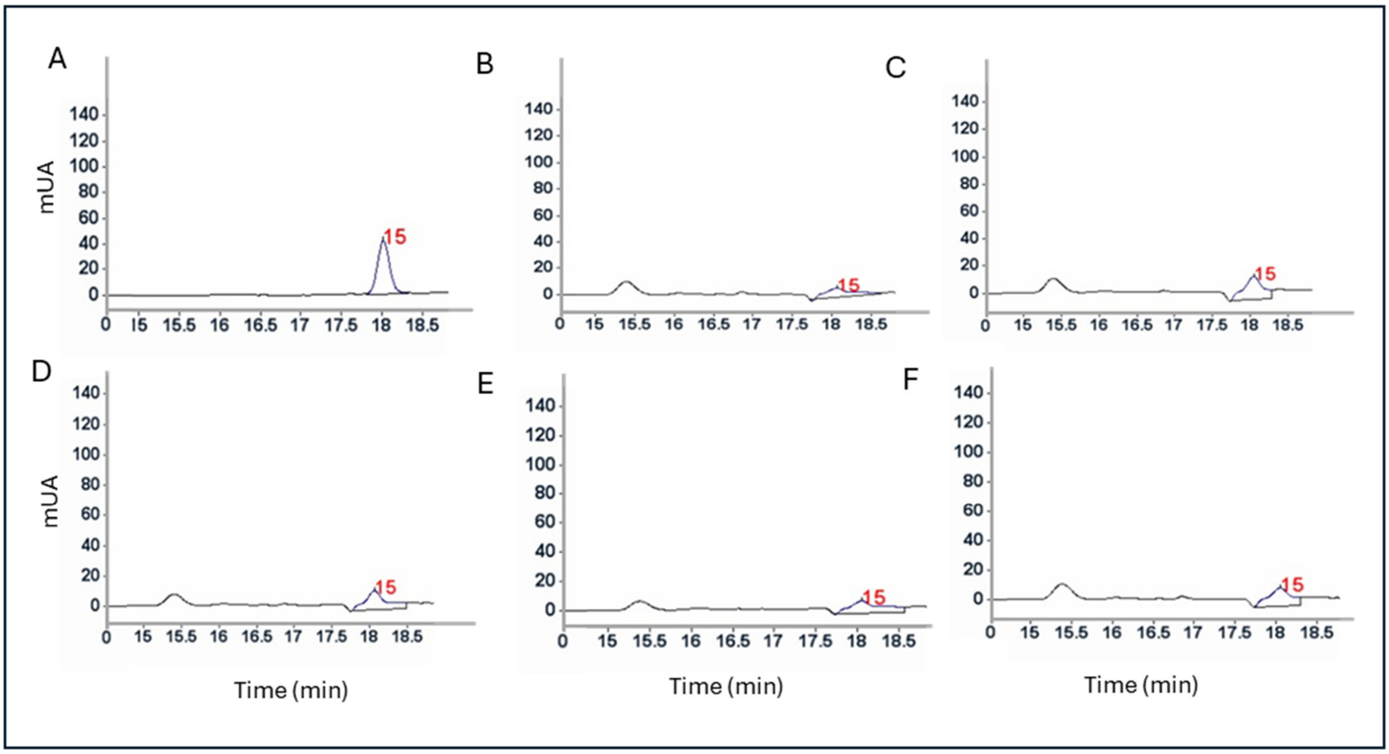

Figure S10. Chromatogram of the amino acid proline recorded at 262 nm. (A) Reference standard; (B) *Gambierdiscus* cf. *caribeaus* strains GbSa-2 and (C) GbSa-7 and *Gambierdiscus* cf. *carpenteri* strains (D) GbSa-4, (E) GbSa-5, and (F) GbSa-6.

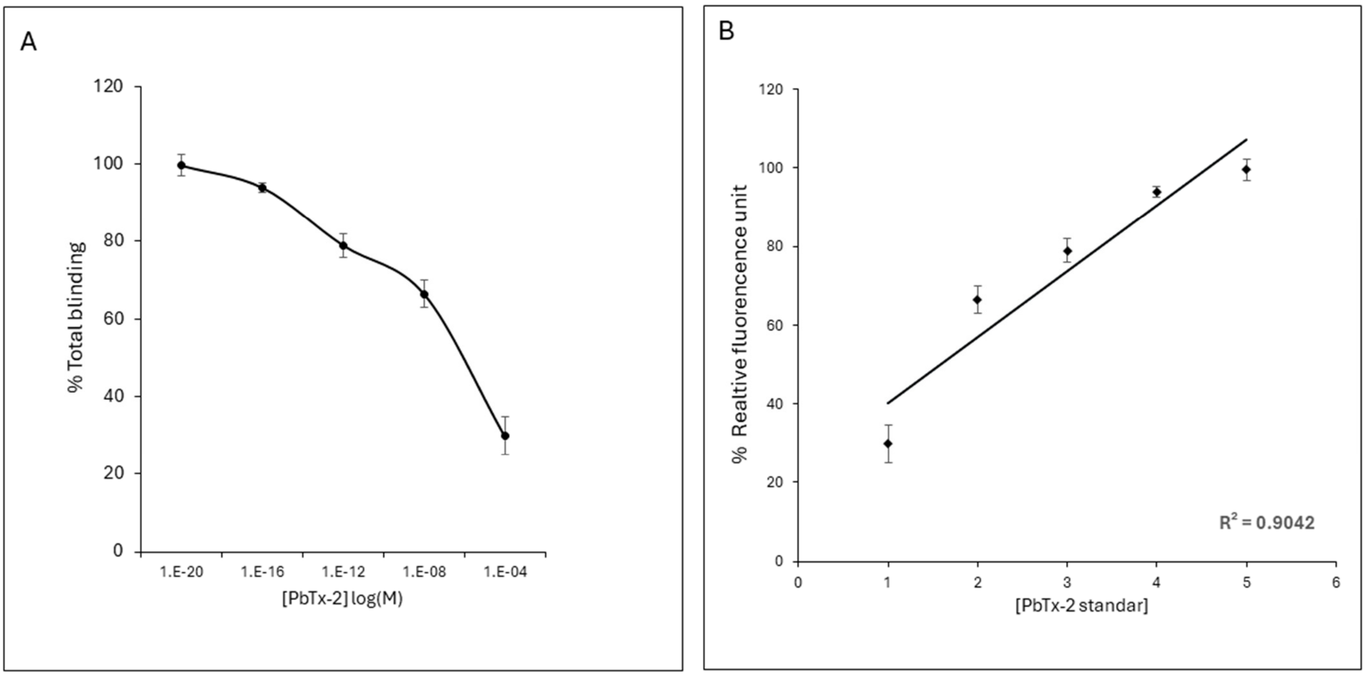

Figure S11. (A) Sigmoid dose response curve as a percentage of total binding. (B) Linear standard curve with concentration in dilution series from brevetoxin standard to extrapolated PbTx equivalents.
